# The evolutionary history of genes involved in spoken and written language: beyond *FOXP2*

**DOI:** 10.1038/srep22157

**Published:** 2016-02-25

**Authors:** Alessandra Mozzi, Diego Forni, Mario Clerici, Uberto Pozzoli, Sara Mascheretti, Franca R. Guerini, Stefania Riva, Nereo Bresolin, Rachele Cagliani, Manuela Sironi

**Affiliations:** 1 Bioinformatics, Scientific Institute IRCCS E. MEDEA, 23842 Bosisio Parini, Italy; 2 Department of Physiopathology and Transplantation, University of Milan, 20090 Milan, Italy; 3 Don C. Gnocchi Foundation ONLUS, IRCCS, 20100 Milan, Italy; 4 Child Psychopathology Unit, Scientific Institute IRCCS E. MEDEA, 23842 Bosisio Parini, Lecco, Italy; 5 Dino Ferrari Centre, Department of Physiopathology and Transplantation, University of Milan, Fondazione Ca’ Granda IRCCS Ospedale Maggiore Policlinico, 20122 Milan, Italy

## Abstract

Humans possess a communication system based on spoken and written language. Other animals can learn vocalization by imitation, but this is not equivalent to human language. Many genes were described to be implicated in language impairment (LI) and developmental dyslexia (DD), but their evolutionary history has not been thoroughly analyzed. Herein we analyzed the evolution of ten genes involved in DD and LI. Results show that the evolutionary history of LI genes for mammals and aves was comparable in vocal-learner species and non-learners. For the human lineage, several sites showing evidence of positive selection were identified in *KIAA0319* and were already present in Neanderthals and Denisovans, suggesting that any phenotypic change they entailed was shared with archaic hominins. Conversely, in *FOXP2*, *ROBO1*, *ROBO2*, and *CNTNAP2* non-coding changes rose to high frequency after the separation from archaic hominins. These variants are promising candidates for association studies in LI and DD.

Language, intended as the capacity to generate a limitless range of expressions using the combination of a limited set of elements and rules, is a distinctive attribute of humans. Other animals, including great apes, communicate using more simple systems that lack the open-ended power of human language^1^. An important component for the development of spoken language is the capacity of imitation. Vocal imitation and learning are not exclusively human, as different species of songbirds, in addition to hummingbirds and parrots, are known to learn vocalization by imitation. These species have often been referred to as “vocal-learners”, although recent observations suggest that vocal-learning abilities may be distributed as a continuum rather than as a categorical trait^2^. Thus, among mammals, some marine (cetaceans, pinnipeds) and terrestrial (elephant and some bats) species may be described as complex-vocal learners^2^,^3^. For the sake of simplicity, herein we refer to complex-vocal learners (both mammalian and avian) as vocal-learners and to all other species as non-learners. To date, the evolutionary origin of complex-vocal learning (independent gains, multiple losses from a complex-vocal learner ancestor, continuum in vocal learning abilities) remain to be elucidated^1^,^2^. However, animal vocalization, including birdsong, lack semantics and syntax, thus differing substantially from human language^4^.

Importantly, whereas most animals use vocal communication, humans are unique in their use of written language. This implies the development of a system of decoding among sounds, symbols, and concepts. As first suggested by Mattingly^5^, *“reading is parasitic on speech”*, as it depends on all the components of the spoken language: syntax, morphology, phonology, pragmatics, and lexicon^6^,^7^.

The close relationship between spoken and written language skills is well accepted and particularly evident in the comorbidity between language impairment (LI) and developmental dyslexia (DD)^8^. LI and DD are common neurodevelopmental disorder characterized by unexpected difficulties with verbal language and reading, respectively, despite adequate educational and socioeconomic opportunity and instruction, as well as otherwise normal development^9^ .

In recent years, molecular genetics studies in family or case-control settings have identified candidate genes for LI and DD, with several genetic risk factors contributing to both conditions^10^,^11^. The first gene to be implicated in a severe speech and language disorder was *FOXP2*, found to be mutated in a large family affected by verbal dyspraxia^12^. Since its identification, the role of *FOXP2* in language (dis)abilities has been independently confirmed in several studies^13^, and an evolutionary analysis of its coding sequence revealed two human-specific amino acid substitutions^14^. This led to the hypothesis that recent changes in the FOXP2 protein have contributed to the development of human verbal skills^14^. This possibility has been supported by studies with animal models and cell lines^15^,^16^, but challenged by other observations^17^,^18^.

The discovery that Neanderthals already possessed the human-specific *FOXP2* variants^17^ fueled speculation on their impact (or lack thereof) on the development of language^1^ and on the timing of modern language origin^19^,^20^. In fact, considerable debate still exists as to whether archaic hominins possessed a communication system comparable to that of modern humans^19^,^20^,^21^,^22^,^23^.

More recently, Maricic and coworkers^24^ identified a regulatory substitution in *FOXP2* that is almost fixed in modern human populations, but absent in Neanderthals and Denisovans. The authors suggested that a combination of coding and regulatory variants in *FOXP2* contributed to the development of modern language. Whereas this possibility remains to be verified, the *FOXP2* example highlights the power of evolutionary analyses to generate specific hypotheses that can be tested using molecular genetics approaches.

After the identification of *FOXP2*, a number of genes have been described to be implicated in LI and DD, but their evolutionary history was not thoroughly examined. Herein we took advantage of genetic diversity data for human populations and great apes, as well as of genomic information for archaic hominins, mammals, and birds to provide insight into the evolution of ten genes involved in LI and DD. These genes were selected based on the evidence of association with LI and/or DD in humans (Table 1). Most of them have established functions in brain processes including neuronal migration, cell adhesion, or axon guidance (*ROBO1, ROBO2, KIAA0319, DYX1C1, CNTNAP2*), as well as calcium homeostasis (*ATP2C2*)^10^,^25^,^26^.

## Results

### Adaptive evolution in Mammals and Aves

We analyzed the evolutionary history of 10 genes reliably associated with LI and DD (Table 1) by retrieving mammalian and avian coding sequences from public databases (see methods; Supplementary Table S1). Recombination can confound evolutionary analyses by introducing apparent substitution rate heterogeneity among sites^27^, and by causing the estimated phylogeny to have excessively long terminal branches^28^. We thus screened the DNA alignments for the presence of recombination with GARD (genetic algorithm recombination detection). In the mammalian phylogeny breakpoints were detected in *CMIP, CNTNAP2, DCDC2*, and *FOXP2*; in aves, one breakpoint was detected for the *DCDC2* gene (Supplementary Table S2). Taking this information into account, we calculated the average non-synonymous substitution/synonymous substitution rate (dN/dS, also referred to as ω) for the ten genes using the single-likelihood ancestor counting (SLAC) method^29^. As observed for most mammalian and avian genes^30^,^31^, the dN/dS ratio was lower than 1 in all cases (Table 1), indicating that purifying selection is the major force shaping diversity at LI and DD genes in both animal classes. Because positive selection can act on a few sites in a protein that is otherwise selectively constrained, we applied likelihood ratio tests (LRT) implemented in the *codeml* program^32^. LRTs were run over whole gene alignment or on subregions split on the basis of the recombination breakpoints. Under two different codon frequency models (F3 × 4 and F61), two neutral models (M8a and M7) were rejected in favor of the M8 positive selection model for the mammalian *ATP2C2, CNTNAP2, DYX1C1, NFXL1*, and *ROBO2* genes (Table 2 and Supplementary Table S3). In aves, these conditions were verified for *ATP2C2, DCDC2, FOXP2*, and *NFXL1* (Table 2 and Supplementary Table S3). Thus, these genes represented targets of positive selection in mammals, birds, or both.

**Figure 1:**
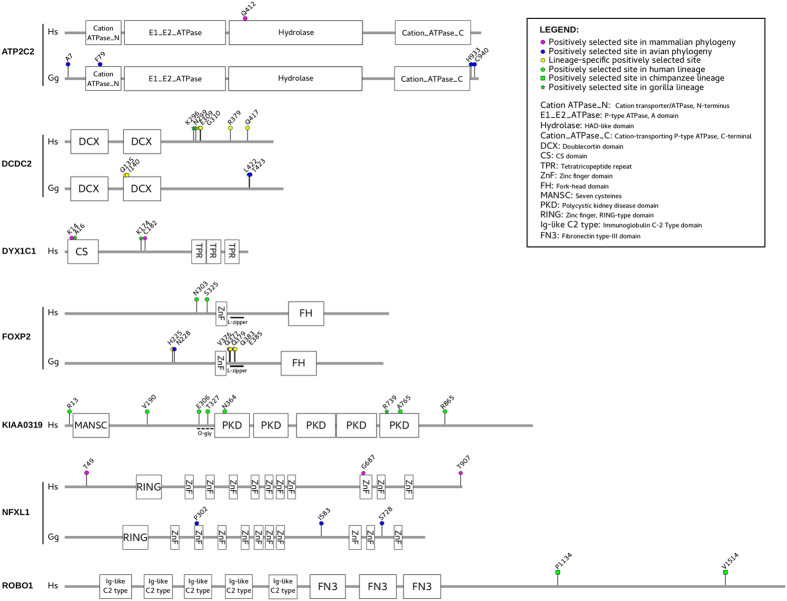
Domain representation of positively selected genes. Sites showing evidence of positive selection are mapped onto the domain representation of the protein. Positions for mammalian and avian genes refer to the human and chicken sequences, respectively (see also Supplementary Table S2) Color codes and domain names are reported.

The Bayes Empirical Bayes (BEB) analysis^33^,^34^ and the Mixed Effects Model of Evolution (MEME)^35^ were next applied to the selected genes in order to identify specific sites targeted by positive selection. To limit false positive results, only sites detected using both methods were considered (Fig. 1 and Table 2).

Among sites showing evidence of positive selection, K14 in mammalian DYX1C1 is located in the CS (or p23) domain, which is involved in the maintenance of folding and in protein-protein interaction^36^ (Fig. 1). The CS domain of DYX1C1 interacts with Hsp70, Hsp90, an E3 ubiquitin ligase known as CHIP^37^, as well as with the estrogen receptors (ERα and Erβ)^38^.

In aves, residue F79 in ATP2C2 is located in the cation ATPase_N domain, which is thought to regulate enzyme function^39^ (Fig. 1).

As for FOXP2, residue Q383 is part of a leucine-zipper region flanking the Zinc-finger domain (Znf) of the forkhead box protein P2 (Fig. 1); generally these regions are functionally required for dimerization and transcriptional regulation^40^.

Finally, in NFXL1, which is believed to act as a transcriptional repressor^41^, two residues showing evidence of positive selection (G687 and P302, in mammals and birds, respectively) are located in the Znf domains, stable finger-like protrusion that make tandem contacts with DNA.

### Lineage-specific selection in mammals and birds

We next extended our analysis to explore possible variations in selective pressure across lineages.

**Figure 2:**
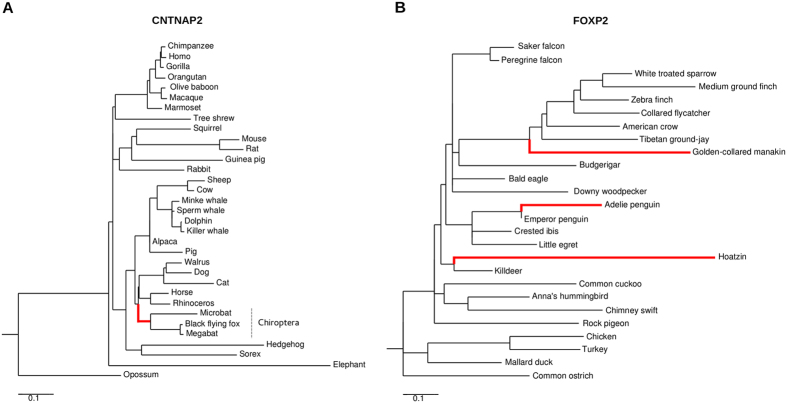
Branch-site analysis of positive selection. aBS-REL analysis for the *CNTNAP2* (**A**) and *FOXP2* (**B**) genes in mammals and birds, respectively. Branch lengths are scaled to the expected number of substitutions per nucleotide. Red: branches that were confirmed to be under episodic positive selection using the *codeml* branch-site models.

Specifically, we aimed to assess whether specific branches in the phylogenetic trees evolved under episodic positive selection. Because we did not want to make any *a priori* assumption about which lineages were more likely to have experienced adaptive evolution, the adaptive branch site-random effects likelihood (aBS-REL) method was applied^42^. Branches identified with aBS-REL were cross-validated using the branch-site LRT models implemented in *codeml*^43^. To be conservative, only branches that were supported by statistical evidence using both methods were considered (Table 3, Figs 1,2 and Supplementary Fig. S1). Positively selected sites for specific lineages were detected using the intersection of the BEB and MEME results.

Overall, evidence of episodic positive selection was obtained for few lineages both in the mammalian and in the bird phylogenies.

No primate lineage or node resulted to have undergone episodic selection at these genes. Previous data^44^ indicated different selective pressure at the *ROBO1* gene for the Homininae (human-chimpanzee-gorilla) branch; however, the branch-site LRT models provided no statistically significant evidence of episodic selection (nor did aBS-REL).

Interestingly, episodic positive selection was detected for the bat branch at the *CNTNAP2* gene (Fig. 2 and Table 3). In aves, three lineages, none of them representing vocal-learner species, showed robust evidence of episodic positive selection at *FOXP2* (Table 3 and Figs 1,2). Most selected sites in avian FOXP2 are located within or in the vicinity of the leucine-zipper motif (Fig. 1).

### Positive selection in humans and great apes

The *FOXP2* gene acquired two amino acid substitutions (N303 and S325) after the split of humans from their common ancestor with chimpanzees^14^,^45^, leading to the suggestion that the two changes might have contributed to the development of human linguistic abilities^14^. The availability of extensive genetic diversity data for humans and great apes now allows more thorough investigation of the evolution of genes involved in the development of human-specific abilities. Thus, we applied a population genetics-phylogenetics approach to analyze the evolutionary pattern of LI as well as DD genes in the human, chimpanzee, and gorilla lineages. In particular, we applied gammaMap^46^ that jointly uses intra-species variation and inter-specific diversity to estimate the distribution of selection coefficients (γ) along coding regions. gammaMap envisages 12 classes of γ, ranging from strongly beneficial (γ = 100) to inviable (γ = −500), with γ equal to 0 indicating neutrality.

**Figure 3:**
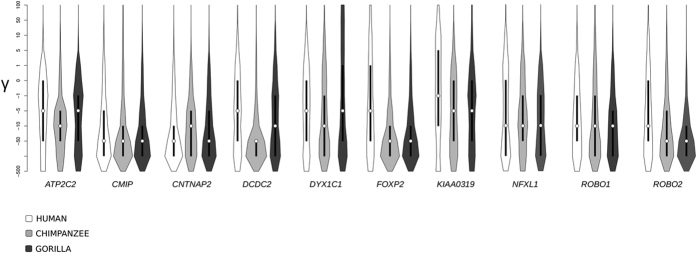
Analysis of selective pressure in the human, chimpanzee and gorilla lineages. Violin plot of selection coefficients for the three primate lineages (median, white dot; interquartile range, black bar). Selection coefficients (γ) are classified as strongly beneficial (100, 50), moderately beneficial (10, 5), weakly beneficial (1), neutral (0), weakly deleterious (−1), moderately deleterious (−5, −10), strongly deleterious (−50, −100), and inviable (−500).

In line with the SLAC results, all genes were found to evolve under some degree of purifying selection (in all cases the median gamma was lower than or equal to – 1) in the three species (Fig. 3). Overall, selection coefficients tended to be lower for gorilla and chimpanzee than for human genes (Fig. 3).

Analysis of sites showing evidence of positive selection (defined as codons with a posterior probability >0.75 of γ ≥1) confirmed N303 and S325 in human FOXP2. In humans, seven sites were also identified in KIAA0319; all of them are located in the extracellular domain of the protein, with the exception of R13, that is part of the signal peptide. In particular, two sites (E306 and T327) fall within the predicted mucin-type O-glycosilation region^47^, and two residues (N364 and V765) are located in the PKD domains. These latter play a role in cell-cell adhesion processes^48^ (Fig. 1). Site 735, showing evidence of positive selection, is polymorphic in humans and corresponds to a low frequency SNP (rs2817191, V735A) (Fig. 1 and Supplementary Table S4).

Notably, five sites in DD genes were found to display evidence of positive selection in the gorilla lineage. One of them is within KIAA0319 and is located in the last PKD domain. The positively selected site in the gorilla *DYX1C1* gene is located in the above-mentioned CS domain (Fig. 1 and Supplementary Table S4).

Finally, in the chimpanzee lineage we detected two sites showing evidence of positive selection in *ROBO1*, a gene associated to both language and reading phenotypes in human population studies (Fig. 1, Table 1 and Supplementary Table S4)^44^,^49^.

### Selective sweeps in modern humans

We finally investigated whether positive selection acted on LI and DD genes during the recent evolutionary history of human populations. Using the 1000 Genomes Phase 1 data for Yoruba (YRI), Europeans (CEU), and Chinese (CHB), we calculated pairwise F_ST_^50^, an estimate of population genetic differentiation, and performed the DIND (Derived Intra-allelic Nucleotide Diversity) test^51^. Statistical significance (in terms of percentile rank) was obtained by deriving empirical distributions. SNPs were considered as positive selection targets if a rank ≥0.99 was obtained for both the F_ST_ and DIND tests in the same population.

As a confirmatory signature (but not in the initial detection of selection targets), we calculated normalized values for Fay and Wu’s H (DH)^52^ in sliding windows along the analyzed genomic regions.

**Figure 4:**
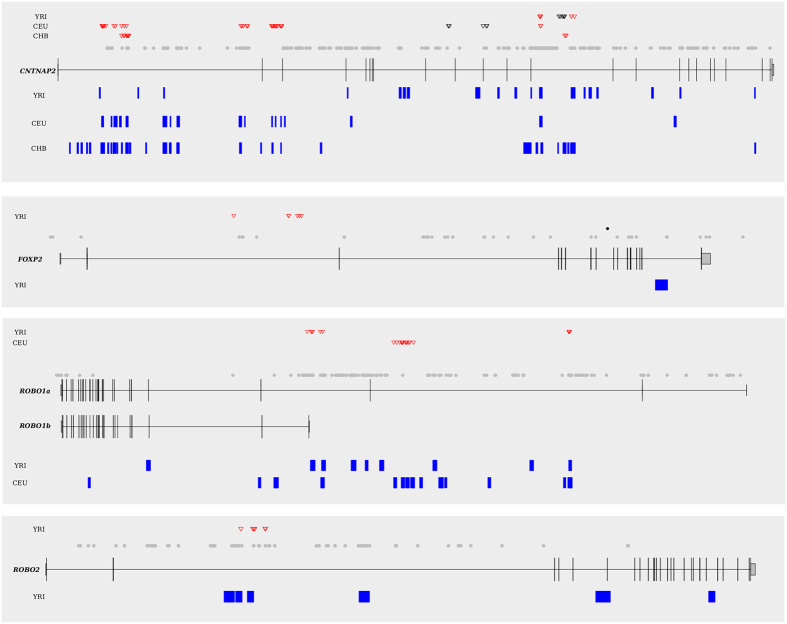
Location of the selection targets in human populations. The gene structures of *CNTNAP2* (**A**), *FOXP2* (**B**), *ROBO1* (**C**), and *ROBO2* (**D**) are shown. Candidate selection targets are shown as triangles, with colors indicating the derived allele frequency of each SNP (red: DAF > 0.80, black: DAF < 0.80). Blue rectangles represent genomic windows with a DH value lower than the 1^th^ percentile (see methods for details). The location of variants cataloged as modern-human-specific sites by^56^ is shown (gray circles). The black dot in (**B**) represents rs114972925 (see text).

Four genes displayed signals of positive selection (Fig. 4 and Supplementary Table S5), with some of them showing multiple signatures possibly ensuing from distinct selective events. Several selective sweeps were accounted for by SNPs that reached high derived allele frequency (DAF) in one or more human populations (Supplementary Table S5); in most cases high DAF signals identified through the F_ST_ and DIND tests were validated by DH (i.e. the DH value was below the 1^st^ percentile), in line with this statistics having maximum power for high-frequency sweeps^52^ (Fig. 4 and Supplementary Table S5). One of the selected haplotypes in *CNTNAP2* carries a set of variants (rs802567, rs802569, rs802571, and rs802558) in full LD (r^2^ = 1 in Europeans) with rs802568, which was associated with schizophrenia and bipolar disorder in genome-wide association studies (the ancestral allele increases disease risk)^53^. A previous population genetics analysis of *FOXP2* targets detected two major selection signatures at the *CNTNAP2* locus^54^. Both signals spatially overlap with those we describe in introns 1 and 13. In *ROBO1*, a cluster of SNPs showing evidence of positive selection surrounds the transcriptional start site of the alternative isoform *ROBO1b* (Fig. 4).

We next investigated whether the selected alleles were already present in archaic hominins.

Analysis of ancient DNA samples indicated that both a Denisova^55^ and an Altai Neandertal^56^ individuals were homozygous for the ancestral allele at the overwhelming majority (86.5%) of SNPs showing evidence of positive selection (Supplementary Table S5). Specifically, all the selected haplotype blocks include a large proportion of alleles unique to modern humans. We thus conclude that all the selective events we detected occurred after the split of modern humans from extinct hominins. In fact, several variants we identified are included in a catalog of modern-human-specific sites- i.e. positions where the Denisova or Altai Neandertal sequences display the ancestral allele, whereas most (>90%) modern humans carry the derived allele^56^ (Fig. 4 and Supplementary Table S5).

Previous analysis of the *FOXP2* gene in Neanderthals indicated that the derived allele at rs114972925 rose to high frequency in modern humans but is absent in archaic hominins (i.e. this variant is a modern-human-specific site)^24^ (Fig. 4). rs114972925 shows very little LD (r^2^ < 0.1 in YRI) with the selection targets we identified in the gene and, using the criteria we applied herein, displays no selection signature (its DIND rank is 0.85 in YRI, DAF is 1 in CEU and CHB).

## Discussion

In this study, we integrated data from different sources to provide a comprehensive analysis of the evolutionary history of genes involved in disorders of spoken and written language. We also performed an analysis of bird species, as these animals are increasingly recognized as excellent models to study the evolution of speech. In fact, vocal-learning species, both mammalian and avian, share specific behavioral and neuronanatomical features ^2^.

We included ten genes in this study, based on the strength of the evidence relating them to either LI, DD or both. Despite their generally strong functional constraint in both mammals and birds, about half of them were found to have evolved under diversifying selection, this latter targeting a small minority of sites in all genes. As we highlight below, because most of these genes are involved in a number of processes and expressed in a variety of tissues, there is no indication that the sites we identified modulate neurocognitive phenotypes in different species. For instance, in both mammals and aves, LI genes were not specifically targeted by episodic positive selection in vocal learning species. For birds, these data are in agreement with a previous study that searched for convergent accelerated evolution in vocal learners compared to non-learners: none of the genes studied herein was identified^31^. The authors, though, tested a specific hypothesis, and the analyses were not devised to detect selection at any lineage or node of the avian phylogeny. We used a different approach, as we did not make any *a priori* assumption. This allowed us to observe significantly higher dN/dS values in *FOXP2* for three non-vocal learner bird species. Most sites showing evidence of positive selection were located in the leucine-zipper motif, a region involved in dimerization. In humans, missense mutations in this region impair FOXP2 transcriptional activity and determine a language deficit phenotype^57^. Nonetheless, because these species lack vocal-learning abilities, it is sensible to conclude that the selective pressure acting on *FOXP2* in these birds is unrelated to vocal communication. This underscores the difficulty of relating individual changes, albeit driven by natural selection, to specific traits across species.

For mammals, no branch in the phylogeny yielded evidence of episodic selection at *FOXP2*. Recently, a higher variability of bat *FOXP2* genes compared to other mammals was reported; this was suggested to be related to echolocation rather than vocal learning^58^. In line with previous results^58^, the branch-site test for the bat lineage was not significant for *FOXP2;* evidence of episodic selection in Chiroptera was instead detected for *CNTNAP2*, a direct transcriptional target of FOXP2. Although bats are regarded as a promising candidate species for studies on vocal production and learning^59^, the results obtained for *FOXP2* in birds should caution against drawing any conclusion about the role of *CNTNAP2* (and *FOXP2*) variability in bats and the evolution of echolocation or vocal learning.

The branch-site tests we applied did not detect lineage-specific selection at *FOXP2* in humans or at *ROBO1* in Homininae. The apparent discrepancy with previous findings lies in the different hypotheses tested: whereas we explicitly tested for positive selection, previous works tested for the constancy of the dN/dS ratio among lineages^14^,^44^. It should also be noted that branch-site tests are robust, but lack power^43^ Indeed, we used gammaMap to search for linage-specific selection in humans and great apes and we detected selection at human *FOXP2*.

Nonetheless, the analysis of the selective patterns of DD and LI genes in the human and great ape lineages needs cautious interpretation. For the human lineage, several sites showing evidence of positive selection were identified in the *KIAA0319* gene, which was repeatedly associated to DD and language abilities^60^,^61^,^62^. Most sites are located in protein regions (the O-glycosilated portion and the PKD domains) potentially involved in cell-cell adhesion and in neuronal migration^48^,^60^. Moreover, two of the selected sites (N364 and R865) are human-specific, meaning that all other mammals sequenced to date carry the same ancestral residue. These substitutions were already present in Neanderthals and Denisovans, suggesting that any phenotypic change they entailed was shared between modern humans and archaic hominins. Furthermore, sites showing evidence of positive selection were detected in the gorilla and chimpanzee lineages at *KIAA0319*, as well as at other genes associated to DD (Fig. 1); three of these sites (E739 in KIAA0319, V16 in DYX1C1, and T296 in DCDC2) are specific to gorillas. Because gorillas cannot read, inference on the nature and effect of selection at these genes remains problematic.

The identification of the two amino acid substitutions in human FOXP2 fostered a number of experimental studies. Introduction of the two human residues in the orthologous mouse protein was shown to determine changes in learning, behavior, as well as in dendrite morphology and synaptic plasticity of cortico-basal ganglia^15^,^63^. Along these lines Konopka and coworkers^16^ showed that human and chimpanzee FOXP2 exert different effects on the transcriptional regulation of neurodevelopmental genes. Thus, despite their being shared with archaic hominins^17^ and, in the case of the N325 site, with carnivores^45^, at least one of the two substitutions is clearly functional and may have an effect on neurodevelopment. An interesting possibility is that several human-specific coding and regulatory changes in genes involved in LI and DD, each contributing relatively subtle effects, account for the development of spoken and written language in modern humans. This hypothesis was also proposed by Marcic and coworkers^24^ upon discovery of a regulatory variant in *FOXP2* that is almost fixed in human populations but absent in Neanderthals. We extended the analysis of recent positive selection in human populations to the ten LI and DD genes. Most selective sweeps we detected are at high-frequency in one or more analyzed populations and all of them occurred after the split of modern humans from archaic hominins. We note that available methods that search for positive selection signals have more power for recent events, and the DIND test applied herein makes no exception^64^. Thus, on one hand, the representation of modern-human-specific alleles among variants detected as selection targets is unsurprising. On the other hand, a number of fixed or almost fixed differences between modern humans and archaic hominins are expected not to be functional and to be due to drift. The combination of selection signals with information from archaic hominin genomes allows the identification of human-specific changes that rose to high frequency through a selective sweep and, therefore, must affect some phenotypic trait, these latter being the targets of natural selection. In fact, one of the signals we identified in *CNTNAP2* is in full LD with a protective allele for schizophrenia and bipolar disorder (rs802568). Although this finding does not necessarily imply that selection primarily acted on the affective disorder phenotype, the selected variant/haplotype does modulate a phenotype. In general, the selective pressure responsible for the detected sweeps may be related to traits distinct from LI and DD, and even from cognitive capacities/disabilities in general. In fact, genome-wide association studies have detected variants in *FOXP2* associated with traits as diverse as IgG glycosylation and blood pressure^65^,^66^. Nonetheless, some signals are particularly suggestive of a functional role in cognitive processes. For example, a cluster of selected SNPs in *ROBO1* surrounds the transcription start site for the *ROBO1b* isoform. *ROBO1a* and *ROBO1b* were shown to be differentially regulated in fetal human brain areas related to hearing and speech^67^,^68^. Adding to the relevance of *ROBO1* transcriptional regulation, Wang *et al*. described its specialized expression in songbird vocal motor cortical regions during critical periods for vocal learning^69^.

Thus, the selected variants/haplotypes we identified represent candidate modifiers of LI and DD phenotypes. In this respect, it is worth mentioning that the minor allele frequency of most selected alleles is very low or zero in several human populations. This is especially true for populations of non African ancestry, which are most often analyzed in genetic studies. Thus, association analysis for these variants will require very large subject samples and/or the recruitment of cohorts of African/mixed African ancestry.

The study of human distinctive traits such as the use of spoken and written language has received enormous attention in the scientific literature. In this field, evolutionary analyses hold the promise to unveil the genetic determinants of human uniqueness. The *FOXP2* case has been epitomal in this respect, highlighting the strengths and weaknesses of evolutionary inference. Data herein extend the analysis to several other genes to generate an overall complex picture, whereby selection signatures are often difficult to relate to specific traits. The selected sites we identified should be regarded as potential modifiers of phenotypic traits, these latter not necessarily related to LI, DD, or other cognitive functions. Experimental analyses will be necessary to address the functional role of the selected changes we report and the phenotype they modulate. The lack of suitable experimental models for the study of human-specific traits, though, will make this task difficult to accomplish.

## Methods

### Gene selection

We analyzed genes that have been reliably associated to language impairment (LI) and developmental dyslexia (DD) (Table 1), as summarized by Paracchini and by Carrion-Castillo *et al*.^10^,^11^. We also included *FOXP2*, known as the “language gene”^12^, as well as *NFXL1* and *ROBO2*, that have recently been described as associated to LI or DD^70^,^71^. Genes that were associate to LI or DD in the context of more complex phenotypes (e.g. *FOXP1*^72^,^73^) were not included in the study.

### Evolutionary analysis in mammals and aves

Mammalian and avian coding sequences were retrieved from the Ensembl (http://www.ensembl.org/index.html) and the National Center for Biotechnology Information (NCBI, http://www.ncbi.nlm.nih.gov) databases. Species were selected to be representative and to include vocal-learners in both classes; we analyzed a comparable number of species for the Mammalian and Avian phylogenies (Supplementary Table S1).

DNA alignments were performed using the RevTrans 2.0 utility^74^ and checked by the use of trimAl (automated1 mode)^75^; subsequently, manual editing was used to correct few misalignments in proximity of small gaps .

All alignments were screened for the presence of recombination breakpoints using GARD, a program that uses phylogenetic incongruence among segments of a sequence alignment to detect the best-fit number and location of recombination breakpoints^76^.

We estimated the average non-synonymous substitution/synonymous substitution rate (ω) using SLAC (Single Likelihood Ancestor Counting)^29^. This method was selected because it allows calculation of average dN/dS (and its confidence intervals) while accounting for recombination.

We used PAML (Phylogenetic Analysis by Maximum Likelihood) analysis to detected positive selection^32^. The *codeml* NSsite models that allow (M8) or disallow (M8a, M7) a class of sites to evolve with ω > 1 were fitted to the data using different codon frequencies model: the F3 × 4 model (codon frequencies estimated from the nucleotide frequencies in the data at each codon site) and the F61 model (frequencies of each of the 61 non-stop codons estimated from the data)^32^. The total tree length for the genes or gene regions we analyzed ranged from 0.65 to 10.33; these values are within an optimal accuracy range for *codeml* sites models^33^. Positively selected sites were identified using two different methods: the Bayes Empirical Bayes (BEB) analysis (with a cutoff of 0.90), which calculates the posterior probability that each codon is from the site class of positive selection (under model M8)^33^, and the Mixed Effects Model of Evolution (MEME) (with the default cutoff of 0.1)^35^, which allows the distribution of ω to vary from site to site and from branch to branch at a site. MEME allows the detection of both pervasive and episodic positive selection and has higher power than methods that assume constant dN/dS across lineages^35^.

In order to identify specific branches with a proportion of sites evolving with ω > 1 (i.e. under episodic positive selection), we used aBS-REL, which applies sequential likelihood ratio tests to identify branches under positive selection^42^. One advantage of BS-REL is that it requires no prior knowledge about which lineages are of interest (i.e. are more likely have experienced episodic diversifying selection). Branches identified using this approach were cross-validated using the branch-site likelihood ratio tests from codeml (the so-called modified model A and model MA1, “test 2”)^43^. In this test, branches are divided *a priori* into foreground (those to be analyzed for positive selection) and background lineages, and a likelihood ratio test is applied to compare a model that allows positive selection on the foreground lineages with a model that does not allow such positive selection. An FDR correction was applied to account for multiple hypothesis testing, as previously suggested^77^. BEB analysis from MA (with a cutoff of 0.90) was used to identify sites that evolved under positive selection on specific lineages.

GARD, MEME, SLAC and aBS-REL analyses were performed either through the DataMonkey server^78^ (http://www.datamonkey.org) or run locally (through the HyPhy suite^79^).

### Population genetics-phylogenetics analysis

We exploited data from the 1000 Genomes Pilot Project (1000G) for Europeans (CEU), Yoruba (YRI), and Chinese plus Japanese (CHBJPT)^80^. For chimpanzees and gorillas, we used SNP information from 25 and 27 individuals, respectively^81^. 1000G data were retrieved from the dedicated website (http://www.1000genomes.org/)^80^.

Ancestral sequences were reconstructed by parsimony from the human, chimpanzee, orangutan and macaque sequences.

Analyses were performed with gammaMap^46^, that evaluates intra-specific variation and inter-specific diversity to estimate, along coding regions, the distribution of selection coefficients (γ). In the analysis, we assumed θ (neutral mutation rate per site), k (transitions/transversions ratio), and T (branch length) to vary among genes following log-normal distributions. For each gene we set the neutral frequencies of non-STOP codons (1/61) and the probability that adjacent codons share the same selection coefficient (p = 0.02). For selection coefficients we considered a uniform Dirichlet distribution with the same prior weight for each selection class. For each gene we run 10,000 iterations with thinning interval of 10 iterations.

To be conservative, we declared a codon to be targeted by positive selection when the cumulative posterior probability of γ ≥ 1 was > 0.75, as suggested^82^.

### Human population genetics analyses

Genotype information from the Phase 1 of the 1000 Genomes Project were retrieved from the dedicated website (http://www.1000genomes.org/)^83^. A set of programs developed in C++ using the GeCo++^84^ and the libsequence^85^ libraries was used to organize SNP genotypes in a MySQL database, and to analyze them according to a specific genomic region.

Genotype information was obtained for the 10 genes; in particular, three human populations with different ancestry were analyzed: Europeans (CEU), Africans (Yoruba ,YRI), and East Asians (Han Chinese in Bejing, CHB). A control set of ~2,000 randomly selected genes was used as a reference set (hereafter referred to as control set). These gene were selected to be longer than 5000 bp and have more than 80% human-outgroup (chimpanzee, orangutan or macaque genomes) aligning bases; orthologous regions in the outgroups were retrieved using the LiftOver tool.

The pairwise F_ST_^50^ and the DIND (Derived Intra-allelic Nucleotide Diversity)^51^ test were calculated for all SNPs mapping to the analyzed genes, as well as for SNPs mapping to the control set. F_ST_ values are not independent from allele frequencies, so we binned variants in 50 classes based on the minor allele frequency (MAF) and calculated F_ST_ empirical distribution for each MAF class using the control set data. The same procedure was applied for the DIND test; thus, we calculated statistical significance by obtaining an empirical distribution of DIND values for variants located within control genes; in particular, the DIND test was calculated using a constant number of 40 flanking variants (20 upstream and 20 downstream), as previously described^86^. DIND values for the three human populations were binned in 100 derived allele frequency (DAF) classes, and for each class the distributions were calculated. As suggested^51^, for values of iπ_D_ = 0 we set the DIND value to the maximum obtained over the corresponding class plus 20. Only SNPs with both F_ST_ and DIND with a percentile rank ≥0.99 were considered as selection targets.

We also calculated DH^52^ as a confirmatory signature of positive section in human populations, using an approach based on 5 kb sliding windows moving with a step of 500 bp. Sliding window analyses have an inherent multiple testing problem that is difficult to correct because of the non-independence of windows. In order to partially account for this limitation, we calculated DH also for the control gene set, and the distribution of the statistic was obtained for the corresponding windows. This allowed calculation of the 1^th^ percentile and the identification of regions below this threshold.

In order to avoid spurious signals of selection, we evaluated the level of linkage disequilibrium (LD) between selected SNPs in the same population, and we defined a SNP as a positive selection target if it showed strong LD (r^2^ > 0.80) with at least other two selected SNPs.

## Additional Information

**How to cite this article**: Mozzi, A. *et al*. The evolutionary history of genes involved in spoken and written language: beyond *FOXP2*. *Sci. Rep*. **6**, 22157; doi: 10.1038/srep22157 (2016).

## Supplementary Material

Supplementary Information

## Figures and Tables

**Table 1:** List of genes.

Gene	Protein name	Mammals, Average dN/dS (CI)	Aves, Average dN/dS (CI)	Disorder^a^	Compromised ability^b^	Key references
*ATP2C2*	Calcium-transporting ATPase type 2C member 2 (ATPase 2C2)	0.114 (0.108, 0.120)	0.102 (0.094, 0.111)	LI	Language	87
*CMIP*	C-Maf-inducing protein (c-Mip)	0.022 (0.018, 0.026)	0.023 (0.017, 0.031)	LI	Language, reading	61,62,87
*CNTNAP2*	Contactin-associated protein-like 2	0.074 (0.070, 0.079)	0.076 (0.069, 0.084)	LI	Language, reading	61,88,89
*DCDC2*	Doublecortin domain-containing protein 2	0.222 (0.207, 0.238)	0.386 (0.361, 0.413)	DD	Reading	62,90,91
*DYX1C1*	Dyslexia susceptibility 1 candidate gene 1 protein	0.228 (0.214, 0.242)	0.361 (0.333, 0.389)	DD	Reading^92^,^93^	92,93
*FOXP2*	Forkhead box protein P2	0.034 (0.028, 0.042)	0.100 (0.080, 0.124)	LI	Language, Speech	12
*KIAA0319*	Dyslexia-associated protein KIAA0319	0.312 (0.301, 0.324)	0.237 (0.222, 0.252)	DD	Reading, language	60,61,62,94
*NFXL1*	NF-X1-type zinc finger protein NFXL1	0.149 (0.141, 0.158)	0.139 (0.128, 0.152)	LI	Language, Speech	70
*ROBO1*	Roundabout homolog 1	0.054 (0.050, 0.058)	0.045 (0.040, 0.050)	DD	Reading, Language	44,49
*ROBO2*	Roundabout homolog 2	0.052 (0.048, 0.057)	0.046 (0.040, 0.052)	DD	Language	71

^a^Disoder initially associated to the gene

^b^Compromised abilities associated to variants in the gene.

**Table 2:** Likelihood ratio test (LRT) statistics for models of variable selective pressure among sites (codon frequency: F3 × 4).

MAMMALS				
GENES	Model^a^	− 2ΔlnL^b^	*p*Value (Bonferroni corrected)	MEME-BEB sites^c^
*ATP2C2*	M8a vs M8	14.825	1.180 × 10^−4^	Q412
	M7 vs M8	11.517	3.156 × 10^−3^	
*CNTNAP2 (reg2)*	M8a vs M8	23.894	1.018 × 10^−6^ (2.036 × 10^−6^)	–
	M7 vs M8	12.165	2.282 × 10^−3^ (4.565 × 10^−3^)	
*DYX1C1*	M8a vs M8	16.610	4.592 × 10^−5^	K14, C182
	M7 vs M8	29.270	4.406 × 10^−7^	
*NFXL1*	M8a vs M8	12.237	4.684 × 10^−4^	T49, G687, T907
	M7 vs M8	71.035	3.757 × 10^−16^	
*ROBO2*	M8a vs M8	4.156	4.148 × 10^−2^	–
	M7 vs M8	32.032	1.107 × 10^−7^	
**AVES**				
**GENES**	**Model^a^**	**− 2ΔlnL^b^**	***p* Value (Bonferroni corrected)**	**MEME-BEB sites^d^**
*ATP2C2*	M8a vs M8	7.878	5.004 × 10^−3^	A7, F79, H933, C940
	M7 vs M8	23.618	7.436 × 10^−6^	
*DCDC2 (reg2)*	M8a vs M8	27.893	1.282 × 10^−7^ (2.564 × 10^−7^)	L422, T423
	M7 vs M8	42.071	7.319 × 10^−10^ (1.464 × 10^−9^)	
*FOXP2*	M8a vs M8	11.320	3.483 × 10^−3^	N228, V376, Q383
	M7 vs M8	8.741	3.112 × 10^−3^	
*NFXL1*	M8a vs M8	17.001	3.735 × 10^−5^	P302, I583, S728
	M7 vs M8	33.257	6.002 × 10^−8^	

Notes:

^a^M7 is a null model that assumes that 0 < ω < 1 is beta distributed among sites; M8 (positive selection model) is the same as M7 but also includes an extra category of sites with ω > 1. M8a is the same as M8, except that the 11^th^ category cannot allow positive selection, but only neutral evolution.

^b^2ΔlnL: twice the difference of the natural logs of the maximum likelihood of the models being compared.

^c^Positions refer to the human sequence (see also Supplementary table 2)

^d^Positions refer to the chicken sequence (see also Supplementary table 2).

**Table 3:** Likelihood ratio test (LRT) statistics for models of variable selective pressure among branches.

MAMMALS				
Gene	Foreground branch (MA versus MA1)^a^	−2lnL^b^	*p*value (FDR corrected)	MEME-BEB Sites^c^
*CNTNAP2, reg1*	Chiroptera	8.948	2.778 × 10^−3^	–
*DCDC2, reg2*	Alpaca	18.546	1.658 × 10^−5^ (1.658 × 10^−5^)	–
	Dolphin	23.572	1.204 × 10^−6^ (2.328 × 10^−6^)	E309, G310
	Ruminantia	23.082	1.552 × 10^−6^ (2.328 × 10^−6^)	R379, Q417
**AVES**				
**Gene**	**Foreground branch (MA versus MA1)^a^**	**−2lnL^b^**	***p* value (FDR corrected)**	**MEME-BEB Sites^d^**
*DCDC2, reg1*	Pigeon	24.931	5.941 × 10^−7^	Q135, I140
*FOXP2*	Hoatzin	47.666	5.054 × 10^−12^ (1.516 × 10^−11^)	Q377, Q379
	Adelie penguin	17.596	2.732 × 10^−5^ (4.098 × 10^−5^)	E385
	Golden-collared manakin	11.781	5.985 × 10^−4^ (5.985 × 10^−4^)	H225
*ROBO2*	Chimney swift	15.853	6.846 × 10^−5^ (1.141 × 10^−4^)	–
	Zebra Finch	36.886	1.252 × 10^−9^ (2.504 × 10^−9^)	A814, A815, S816,T817

Notes:

^a^MA and MA1 are branch-site models that assume four classes of sites: the MA model allows a proportion of codons to have ω ≥ 1 on the foreground branches, whereas the MA1 model does not.

^b^2ΔlnL: twice the difference of the natural logs of the maximum likelihood of the models being compared.

^c^Positions refer to the human sequence (see also Supplementary table 2)

^d^Positions refer to the chicken sequence (see also Supplementary table 2).

## References

[b1] Scharff C. & Petri J. Evo-devo, deep homology and FoxP2: implications for the evolution of speech and language. Philos. Trans. R. Soc. Lond. B. Biol. Sci. 366, 2124–2140; doi: 10.1098/rstb.2011.0001 (2011). 21690130 PMC3130369

[b2] Petkov C. I. & Jarvis E. D. Birds, primates, and spoken language origins: behavioral phenotypes and neurobiological substrates. Front. Evol. Neurosci. 4, 12; doi: 10.3389/fnevo.2012.00012 (2012). 22912615 PMC3419981

[b3] Arriaga G. & Jarvis E. D. Mouse vocal communication system: are ultrasounds learned or innate? Brain Lang. 124, 96–116; doi: 10.1016/j.bandl.2012.10.002 (2013). 23295209 PMC3886250

[b4] Berwick R. C., Okanoya K., Beckers G. J. & Bolhuis J. J. Songs to syntax: the linguistics of birdsong. Trends Cogn. Sci. 15, 113–121; doi: 10.1016/j.tics.2011.01.002 (2011). 21296608

[b5] Mattingly I. G. Reading, the linguistic process and linguistic awareness. In Language by ear and by eyes: the relationships between speech and reading (ed. Kavanagh J. F., Mattingly I. G.) 133–147 (Cambridge, MA: MIT Press, 1972).

[b6] van der Lely H. K. & Pinker S. The biological basis of language: insight from developmental grammatical impairments. Trends Cogn. Sci. 18, 586–595; doi: 10.1016/j.tics.2014.07.001 (2014). 25172525

[b7] Hulme C. & Snowling M. J. The interface between spoken and written language: developmental disorders. Philos. Trans. R. Soc. Lond. B. Biol. Sci. 369, 20120395; doi: 10.1098/rstb.2012.0395 (2013). 24324239 PMC3866425

[b8] Peterson R. L. & Pennington B. F. Developmental dyslexia. Lancet 379, 1997–2007; doi: 10.1016/S0140-6736(12)60198-6 (2012). 22513218 PMC3465717

[b9] American Psychiatric Association & American Psychiatric Association. Diagnostic and statistical manual of mental disorders (DSM). Washington, DC: American psychiatric association. 143–147 (1994).

[b10] Carrion-Castillo A., Franke B. & Fisher S. E. Molecular genetics of dyslexia: an overview. Dyslexia 19, 214–240; doi: 10.1002/dys.1464 (2013). 24133036

[b11] Paracchini S. Dissection of genetic associations with language-related traits in population-based cohorts. J. Neurodev Disord. 3, 365–373; doi: 10.1007/s11689-011-9091-6 (2011). 21894572 PMC3230763

[b12] Lai C. S., Fisher S. E., Hurst J. A., Vargha-Khadem F. & Monaco A. P. A forkhead-domain gene is mutated in a severe speech and language disorder. Nature 413, 519–523; doi: 10.1038/35097076 (2001). 11586359

[b13] Graham S. A. & Fisher S. E. Decoding the genetics of speech and language. Curr. Opin. Neurobiol. 23, 43–51; doi: 10.1016/j.conb.2012.11.006 (2013). 23228431

[b14] Enard W. . Molecular evolution of FOXP2, a gene involved in speech and language. Nature 418, 869–872; doi: 10.1038/nature01025 (2002). 12192408

[b15] Enard W. FOXP2 and the role of cortico-basal ganglia circuits in speech and language evolution. Curr. Opin. Neurobiol. 21, 415–424; doi: 10.1016/j.conb.2011.04.008 (2011). 21592779

[b16] Konopka G. . Human-specific transcriptional regulation of CNS development genes by FOXP2. Nature 462, 213–217; doi: 10.1038/nature08549 (2009). 19907493 PMC2778075

[b17] Krause J. . The derived FOXP2 variant of modern humans was shared with Neandertals. Curr. Biol. 17, 1908–1912; doi: 10.1016/j.cub.2007.10.008 (2007). 17949978

[b18] Janik V. M. Cetacean vocal learning and communication. Curr. Opin. Neurobiol. 28, 60–65; doi: 10.1016/j.conb.2014.06.010 (2014). 25057816

[b19] Ackermann H., Hage S. R. & Ziegler W. Brain mechanisms of acoustic communication in humans and nonhuman primates: an evolutionary perspective. Behav. Brain Sci. 37, 529–546; doi: 10.1017/S0140525X13003099 (2014). 24827156

[b20] Johansson S. Neanderthals did speak, but FOXP2 doesn’t prove it. Behav. Brain Sci. 37, 558-9; discussion 577–604; doi: 10.1017/S0140525X13004068 (2014). 25514948

[b21] Dediu D. & Levinson S. C. On the antiquity of language: the reinterpretation of Neandertal linguistic capacities and its consequences. Front. Psychol. 4, 397; doi: 10.3389/fpsyg.2013.00397 (2013). 23847571 PMC3701805

[b22] Berwick R. C., Friederici A. D., Chomsky N. & Bolhuis J. J. Evolution, brain, and the nature of language. Trends Cogn. Sci. 17, 89–98; doi: 10.1016/j.tics.2012.12.002 (2013). 23313359

[b23] Berwick R. C., Hauser M. D. & Tattersall I. Neanderthal language? Just-so stories take center stage. Front. Psychol. 4, 671; doi: 10.3389/fpsyg.2013.00671 (2013). 24069017 PMC3781312

[b24] Maricic T. . A recent evolutionary change affects a regulatory element in the human FOXP2 gene. Mol. Biol. Evol. 30, 844–852; doi: 10.1093/molbev/mss271 (2013). 23197593

[b25] Long H. . Conserved roles for Slit and Robo proteins in midline commissural axon guidance. Neuron 42, 213–223 (2004). 15091338 10.1016/s0896-6273(04)00179-5

[b26] Newbury D. F. & Monaco A. P. Genetic advances in the study of speech and language disorders. Neuron 68, 309–320; doi: 10.1016/j.neuron.2010.10.001 (2010). 20955937 PMC2977079

[b27] Worobey M. A novel approach to detecting and measuring recombination: new insights into evolution in viruses, bacteria, and mitochondria. Mol. Biol. Evol. 18, 1425–1434 (2001). 11470833 10.1093/oxfordjournals.molbev.a003928

[b28] Schierup M. H. & Hein J. Consequences of recombination on traditional phylogenetic analysis. Genetics 156, 879–891 (2000). 11014833 10.1093/genetics/156.2.879 PMC1461297

[b29] Kosakovsky Pond S. L. & Frost S. D. Not so different after all: a comparison of methods for detecting amino acid sites under selection. Mol. Biol. Evol. 22, 1208–1222; doi: 10.1093/molbev/msi105 (2005). 15703242

[b30] Sironi M., Cagliani R., Forni D. & Clerici M. Evolutionary insights into host-pathogen interactions from mammalian sequence data. Nat. Rev. Genet. 16, 224–236; doi: 10.1038/nrg3905 (2015). 25783448 PMC7096838

[b31] Zhang G. . Comparative genomics reveals insights into avian genome evolution and adaptation. Science 346, 1311–1320; doi: 10.1126/science.1251385 (2014). 25504712 PMC4390078

[b32] Yang Z. PAML 4: phylogenetic analysis by maximum likelihood. Mol. Biol. Evol. 24, 1586–1591; doi: 10.1093/molbev/msm088 (2007). 17483113

[b33] Anisimova M., Bielawski J. P. & Yang Z. Accuracy and power of bayes prediction of amino acid sites under positive selection. Mol. Biol. Evol. 19, 950–958 (2002). 12032251 10.1093/oxfordjournals.molbev.a004152

[b34] Yang Z., Wong W. S. & Nielsen R. Bayes empirical bayes inference of amino acid sites under positive selection. Mol. Biol. Evol. 22, 1107–1118; doi: 10.1093/molbev/msi097 (2005). 15689528

[b35] Murrell B. . Detecting individual sites subject to episodic diversifying selection. PLoS Genet. 8, e1002764; doi: 10.1371/journal.pgen.1002764 (2012). 22807683 PMC3395634

[b36] Garcia-Ranea J. A., Mirey G., Camonis J. & Valencia A. p23 and HSP20/alpha-crystallin proteins define a conserved sequence domain present in other eukaryotic protein families. FEBS Lett. 529, 162–167; doi: S0014579302033215 (2002). 12372593 10.1016/s0014-5793(02)03321-5

[b37] Hatakeyama S., Matsumoto M., Yada M. & Nakayama K. I. Interaction of U-box-type ubiquitin-protein ligases (E3s) with molecular chaperones. Genes Cells 9, 533–548; doi: 10.1111/j.1356-9597.2004.00742.x (2004). 15189447

[b38] Massinen S. . Functional interaction of DYX1C1 with estrogen receptors suggests involvement of hormonal pathways in dyslexia. Hum. Mol. Genet. 18, 2802–2812; doi: 10.1093/hmg/ddp215 (2009). 19423554

[b39] Cross R. L. & Muller V. The evolution of A-, F-, and V-type ATP synthases and ATPases: reversals in function and changes in the H+/ATP coupling ratio. FEBS Lett. 576, 1–4; doi: S0014579304010841 (2004). 15473999 10.1016/j.febslet.2004.08.065

[b40] Li S., Weidenfeld J. & Morrisey E. E. Transcriptional and DNA binding activity of the Foxp1/2/4 family is modulated by heterotypic and homotypic protein interactions. Mol. Cell. Biol. 24, 809–822 (2004). 14701752 10.1128/MCB.24.2.809-822.2004 PMC343786

[b41] Mussig C., Schroder F., Usadel B. & Lisso J. Structure and putative function of NFX1-like proteins in plants. Plant. Biol. (Stuttg) 12, 381–394; doi: 10.1111/j.1438-8677.2009.00303.x (2010). 20522174

[b42] Smith M. D. . Less is more: an adaptive branch-site random effects model for efficient detection of episodic diversifying selection. Mol. Biol. Evol. 32, 1342–1353; doi: 10.1093/molbev/msv022 (2015). 25697341 PMC4408413

[b43] Zhang J., Nielsen R. & Yang Z. Evaluation of an improved branch-site likelihood method for detecting positive selection at the molecular level. Mol. Biol. Evol. 22, 2472–2479; doi: 10.1093/molbev/msi237 (2005). 16107592

[b44] Hannula-Jouppi K. . The axon guidance receptor gene ROBO1 is a candidate gene for developmental dyslexia. PLoS Genet. 1, e50; doi: 10.1371/journal.pgen.0010050 (2005). 16254601 PMC1270007

[b45] Zhang J., Webb D. M. & Podlaha O. Accelerated protein evolution and origins of human-specific features: Foxp2 as an example. Genetics 162, 1825–1835 (2002). 12524352 10.1093/genetics/162.4.1825 PMC1462353

[b46] Wilson D. J., Hernandez R. D., Andolfatto P. & Przeworski M. A population genetics-phylogenetics approach to inferring natural selection in coding sequences. PLoS Genet. 7, e1002395; doi: 10.1371/journal.pgen.1002395 (2011). 22144911 PMC3228810

[b47] Velayos-Baeza A., Toma C., Paracchini S. & Monaco A. P. The dyslexia-associated gene KIAA0319 encodes highly N- and O-glycosylated plasma membrane and secreted isoforms. Hum. Mol. Genet. 17, 859–871; doi: 10.1093/hmg/ddm358 (2008). 18063668

[b48] Ibraghimov-Beskrovnaya O. . Strong homophilic interactions of the Ig-like domains of polycystin-1, the protein product of an autosomal dominant polycystic kidney disease gene, PKD1. Hum. Mol. Genet. 9, 1641–1649; doi: 10.1093/hmg/9.11.1641 (2000). 10861291

[b49] Bates T. C. . Genetic variance in a component of the language acquisition device: ROBO1 polymorphisms associated with phonological buffer deficits. Behav. Genet. 41, 50–57; doi: 10.1007/s10519-010-9402-9 (2011). 20949370

[b50] Wright S. Genetical structure of populations. Nature 166, 247–249; doi: 10.1038/166247a0 (1950). 15439261

[b51] Barreiro L. B. . Evolutionary dynamics of human Toll-like receptors and their different contributions to host defense. PLoS Genet. 5, e1000562; doi: 10.1371/journal.pgen.1000562 (2009). 19609346 PMC2702086

[b52] Zeng K., Fu Y. X., Shi S. & Wu C. I. Statistical tests for detecting positive selection by utilizing high-frequency variants. Genetics 174, 1431–1439; doi: 10.1534/genetics.106.061432 (2006). 16951063 PMC1667063

[b53] Wang K. S., Liu X. F. & Aragam N. A genome-wide meta-analysis identifies novel loci associated with schizophrenia and bipolar disorder. Schizophr. Res. 124, 192–199; doi: 10.1016/j.schres.2010.09.002 (2010). 20889312

[b54] Ayub Q. . FOXP2 targets show evidence of positive selection in European populations. Am. J. Hum. Genet. 92, 696–706; doi: 10.1016/j.ajhg.2013.03.019 (2013). 23602712 PMC3644635

[b55] Meyer M. . A high-coverage genome sequence from an archaic Denisovan individual. Science 338, 222–226; doi: 10.1126/science.1224344 (2012). 22936568 PMC3617501

[b56] Prufer K. . The complete genome sequence of a Neanderthal from the Altai Mountains. Nature 505, 43–49; doi: 10.1038/nature12886 (2014). 24352235 PMC4031459

[b57] Roll P. . Molecular networks implicated in speech-related disorders: FOXP2 regulates the SRPX2/uPAR complex. Hum. Mol. Genet. 19, 4848–4860; doi: 10.1093/hmg/ddq415 (2010). 20858596 PMC2989892

[b58] Li G., Wang J., Rossiter S. J., Jones G. & Zhang S. Accelerated FoxP2 evolution in echolocating bats. PLoS One 2, e900: doi: 10.1371/journal.pone.0000900 (2007). 17878935 PMC1976393

[b59] Knornschild M. Vocal production learning in bats. Curr. Opin. Neurobiol. 28, 80–85; doi: 10.1016/j.conb.2014.06.014 (2014). 25050812

[b60] Paracchini S. . The chromosome 6p22 haplotype associated with dyslexia reduces the expression of KIAA0319, a novel gene involved in neuronal migration. Hum. Mol. Genet. 15, 1659–1666; doi: 10.1093/hmg/ddl089 (2006). 16600991

[b61] Newbury D. F. . Investigation of dyslexia and SLI risk variants in reading- and language-impaired subjects. Behav. Genet. 41, 90–104; doi: 10.1007/s10519-010-9424-3 (2011). 21165691 PMC3029677

[b62] Scerri T. S. . DCDC2, KIAA0319 and CMIP are associated with reading-related traits. Biol. Psychiatry 70, 237–245; doi: 10.1016/j.biopsych.2011.02.005 (2011). 21457949 PMC3139836

[b63] Schreiweis C. . Humanized Foxp2 accelerates learning by enhancing transitions from declarative to procedural performance. Proc. Natl. Acad. Sci. USA 111, 14253–14258; doi: 10.1073/pnas.1414542111 (2014). 25225386 PMC4191787

[b64] Fagny M. . Exploring the occurrence of classic selective sweeps in humans using whole-genome sequencing data sets. Mol. Biol. Evol. 31, 1850–1868; doi: 10.1093/molbev/msu118 (2014). 24694833

[b65] Lauc G. . Loci associated with N-glycosylation of human immunoglobulin G show pleiotropy with autoimmune diseases and haematological cancers. PLoS Genet. 9, e1003225; doi: 10.1371/journal.pgen.1003225 (2013). 23382691 PMC3561084

[b66] Sung Y. J., de Las Fuentes L., Schwander K. L., Simino J. & Rao D. C. Gene-smoking interactions identify several novel blood pressure loci in the Framingham Heart Study. Am. J. Hypertens. 28, 343–354; doi: 10.1093/ajh/hpu149 (2015). 25189868 PMC4402348

[b67] Johnson M. B. . Functional and evolutionary insights into human brain development through global transcriptome analysis. Neuron 62, 494–509; doi: 10.1016/j.neuron.2009.03.027 (2009). 19477152 PMC2739738

[b68] Boeckx C. & Benitez-Burraco A. Globularity and language-readiness: generating new predictions by expanding the set of genes of interest. Front. Psychol. 5, 1324; doi: 10.3389/fpsyg.2014.01324 (2014). 25505436 PMC4243498

[b69] Wang R. . Convergent differential regulation of SLIT-ROBO axon guidance genes in the brains of vocal learners. J. Comp. Neurol. 523, 892–906; doi: 10.1002/cne.23719 (2015). 25424606 PMC4329046

[b70] Villanueva P. . Exome sequencing in an admixed isolated population indicates NFXL1 variants confer a risk for specific language impairment. PLoS Genet. 11, e1004925; doi: 10.1371/journal.pgen.1004925 (2015). 25781923 PMC4363375

[b71] St Pourcain B. . Common variation near ROBO2 is associated with expressive vocabulary in infancy. Nat. Commun. 5, 4831; doi: 10.1038/ncomms5831 (2014). 25226531 PMC4175587

[b72] Hamdan F. F. . De novo mutations in FOXP1 in cases with intellectual disability, autism, and language impairment. Am. J. Hum. Genet. 87, 671–678; doi: 10.1016/j.ajhg.2010.09.017 (2010). 20950788 PMC2978954

[b73] Sollis E. . Identification and functional characterization of de novo FOXP1 variants provides novel insights into the etiology of neurodevelopmental disorder. Hum. Mol. Genet ; doi: 10.1093/hmg/ddv495 (2015). 26647308

[b74] Wernersson R. & Pedersen A. G. RevTrans: Multiple alignment of coding DNA from aligned amino acid sequences. Nucleic Acids Res. 31, 3537–3539; doi: 10.1093/nar/gkg609 (2003). 12824361 PMC169015

[b75] Capella-Gutierrez S., Silla-Martinez J. M. & Gabaldon T. trimAl: a tool for automated alignment trimming in large-scale phylogenetic analyses. Bioinformatics 25, 1972–1973; doi: 10.1093/bioinformatics/btp348 (2009). 19505945 PMC2712344

[b76] Kosakovsky Pond S. L., Posada D., Gravenor M. B., Woelk C. H. & Frost S. D. Automated phylogenetic detection of recombination using a genetic algorithm. Mol. Biol. Evol. 23, 1891–1901; doi: 10.1093/molbev/msl051 (2006). 16818476

[b77] Anisimova M. & Yang Z. Multiple hypothesis testing to detect lineages under positive selection that affects only a few sites. Mol. Biol. Evol. 24, 1219–1228; doi: 10.1093/molbev/msm042 (2007). 17339634

[b78] Delport W., Poon A. F., Frost S. D. & Kosakovsky Pond S. L. Datamonkey 2010: a suite of phylogenetic analysis tools for evolutionary biology. Bioinformatics 26, 2455–2457; doi: 10.1093/bioinformatics/btq429 (2010). 20671151 PMC2944195

[b79] Pond S. L., Frost S. D. & Muse S. V. HyPhy: hypothesis testing using phylogenies. Bioinformatics 21, 676–679; doi: 10.1093/bioinformatics/bti079 (2005). 15509596

[b80] 1000 Genomes Project Consortium . A map of human genome variation from population-scale sequencing. Nature 467, 1061–1073; doi: 10.1038/nature09534 (2010). 20981092 PMC3042601

[b81] Prado-Martinez J. . Great ape genetic diversity and population history. Nature 499, 471–475; doi: 10.1038/nature12228 (2013). 23823723 PMC3822165

[b82] Quach H. . Different selective pressures shape the evolution of Toll-like receptors in human and African great ape populations. Hum. Mol. Genet. 22, 4829–4840; doi: 10.1093/hmg/ddt335 (2013). 23851028 PMC3820138

[b83] 1000 Genomes Project Consortium . An integrated map of genetic variation from 1,092 human genomes. Nature 491, 56–65; doi: 10.1038/nature11632; 10.1038/nature11632; (2012). 23128226 PMC3498066

[b84] Cereda M., Sironi M., Cavalleri M. & Pozzoli U. GeCo++: a C++ library for genomic features computation and annotation in the presence of variants. Bioinformatics 27, 1313–1315; doi: 10.1093/bioinformatics/btr123 (2011). 21398667

[b85] Thornton K. Libsequence: a C++ class library for evolutionary genetic analysis. Bioinformatics 19, 2325–2327; doi: 10.1093/bioinformatics/btg316 (2003). 14630667

[b86] Forni D. . An Evolutionary Analysis of Antigen Processing and Presentation across Different Timescales Reveals Pervasive Selection. PLoS Genet. 10, e1004189; doi: 10.1371/journal.pgen.1004189 (2014). 24675550 PMC3967941

[b87] Newbury D. F. . CMIP and ATP2C2 modulate phonological short-term memory in language impairment. Am. J. Hum. Genet. 85, 264–272; doi: 10.1016/j.ajhg.2009.07.004 (2009). 19646677 PMC2725236

[b88] Vernes S. C. . A functional genetic link between distinct developmental language disorders. N. Engl. J. Med. 359, 2337–2345; doi: 10.1056/NEJMoa0802828 (2008). 18987363 PMC2756409

[b89] Whitehouse A. J., Bishop D. V., Ang Q. W., Pennell C. E. & Fisher S. E. CNTNAP2 variants affect early language development in the general population. Genes Brain Behav. 10, 451–456; doi: 10.1111/j.1601-183X.2011.00684.x (2011). 21310003 PMC3130139

[b90] Deffenbacher K. E. . Refinement of the 6p21.3 quantitative trait locus influencing dyslexia: linkage and association analyses. Hum. Genet. 115, 128–138; doi: 10.1007/s00439-004-1126-6 (2004). 15138886

[b91] Schumacher J. . Strong genetic evidence of DCDC2 as a susceptibility gene for dyslexia. Am. J. Hum. Genet. 78, 52–62; doi: 10.1086/498992 (2006). 16385449 PMC1380223

[b92] Taipale M. . A candidate gene for developmental dyslexia encodes a nuclear tetratricopeptide repeat domain protein dynamically regulated in brain. Proc. Natl. Acad. Sci. USA 100, 11553–11558; doi: 10.1073/pnas.1833911100 (2003). 12954984 PMC208796

[b93] Paracchini S. . Analysis of dyslexia candidate genes in the Raine cohort representing the general Australian population. Genes Brain Behav. 10, 158–165; doi: 10.1111/j.1601-183X.2010.00651.x (2011). 20846247 PMC3084500

[b94] Francks C. . A 77-kilobase region of chromosome 6p22.2 is associated with dyslexia in families from the United Kingdom and from the United States. Am. J. Hum. Genet. 75, 1046–1058; doi: 10.1086/426404 (2004). 15514892 PMC1182140

