# Supplementary material for: The evolutionary history of genes involved in spoken and written language: beyond FOXP2
Source: Sci Rep. 2016 Feb 25;6:22157. doi: 10.1038/srep22157 (PMC4766443; doi:10.1038/srep22157)
Supplement: Supplementary Information [file srep22157-s1.pdf]

The evolutionary history of genes involved in spoken and written language: beyond *FOXP2*

Alessandra Mozzi[1*], Diego Forni[1], Mario Clerici[2,3], Uberto Pozzoli[1], Sara Mascheretti[4], Franca R. Guerini[3], Stefania Riva[1], Nereo Bresolin[1,5], Rachele Cagliani[1], and Manuela Sironi[1]

[1] Bioinformatics, Scientific Institute IRCCS E. MEDEA, 23842 Bosisio Parini, Italy.

[2]Department of Physiopathology and Transplantation, University of Milan, 20090 Milan, Italy.

[3]Don C. Gnocchi Foundation ONLUS, IRCCS, 20100 Milan, Italy.

[4]Child Psychopathology Unit, Scientific Institute IRCCS E. MEDEA, 23842 Bosisio Parini, Lecco, Italy.

[5]Dino Ferrari Centre, Department of Physiopathology and Transplantation, University of Milan, Fondazione Ca' Granda IRCCS Ospedale Maggiore Policlinico, 20122 Milan, Italy.

***Corresponding author:** Alessandra Mozzi, PhD, Bioinformatics - Scientific Institute IRCCS E.MEDEA, 23842 Bosisio Parini, Italy. Tel: +39-031877826; Fax:+39-031877499; e-mail: alessandra.mozzi@bp.lnf.it

Supplementary Figure S1. **Branch-site analysis of positive selection**.

aBS-REL analysis for *DCDC2* region 2 in mammals (**A**), *DCDC2* region 1 in birds (**B**), and *ROBO2* in birds (**C**). Branch lengths are scaled to the expected number of substitutions per nucleotide. Red: branches that were confirmed to be under episodic positive selection using the *codeml* branch-site models.

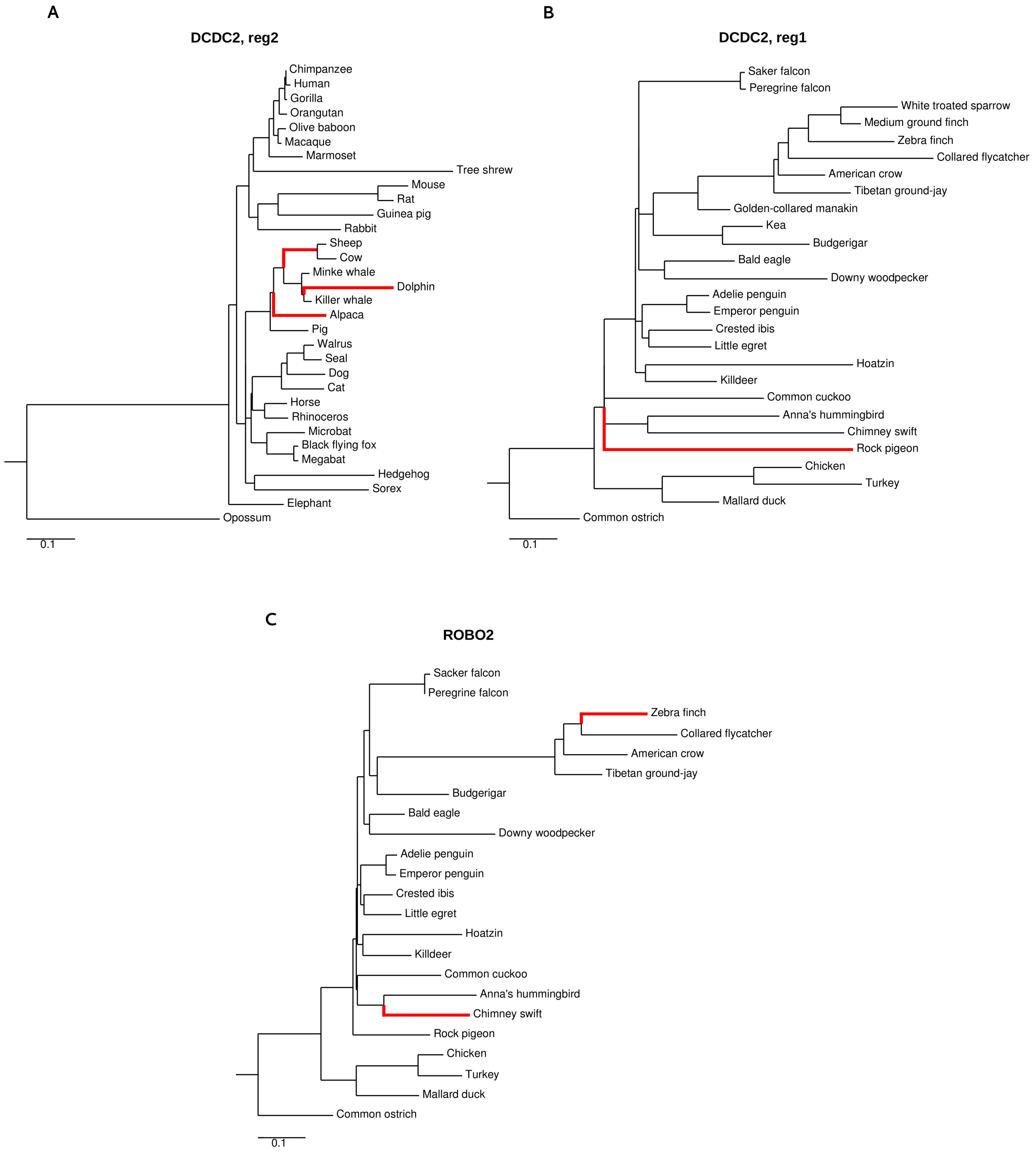

Supplementary Table S1. List of mammalian and avian species

MAMMALS

Common Name	*Scientific Name*	*ATP2C2*	*CMIP*	*CNTNAP2*	*DCDC2*	*DYX1C1*	*FOXP2*	*KIAA0319*	*NFXL1*	*ROBO1*	*ROBO2*
Alpaca	*Vicugna pacos*	■	■	■	■	■	■	■	■	■	■
Black Flying Fox (*)	*Pteropus alecto*	■	■	■	■	■	■		■	■	■
Cat	*Felis catus*	■	■	■	■	■	■	■	■	■	■
Chimpanzee	*Pan troglodytes*	■	■	■	■	■	■	■	■	■	■
Chinese Tree Shrew	*Tupaia chinensis*	■	■	■	■	■	■		■	■	■
Cow	*Bos taurus*	■	■	■	■	■	■	■	■	■	■
Dog	*Canis lupus familiaris*	■	■	■	■	■	■	■	■	■	■
Dolphin *	*Tursiops truncatus*	■	■	■	■	■	■	■	■	■	■
Elephant *	*Loxodonta africana*	■	■	■	■	■	■	■	■	■	■
Gorilla	*Gorilla gorilla gorilla*	■	■	■	■	■	■	■	■	■	■
Guinea Pig	*Cavia porcellus*	■	■	■	■	■	■	■	■	■	■
Hedgehog	*Erinaceus europaeus*	■	■	■	■	■	■	■	■	■	■
Horse	*Equus caballus*	■	■	■	■	■	■	■	■	■	■
Human *	*Homo sapiens*	■	■	■	■	■	■	■	■	■	■
Killer whale *	*Orcinus orca*	■	■	■	■	■	■	■	■	■	■
Macaque	*Macaca mulatta*	■	■	■	■	■	■	■	■	■	■
Marmoset	*Callithrix jacchus*	■	■	■	■	■	■	■	■	■	■
Megabat (*)	*Pteropus vampyrus*	■	■	■	■	■	■		■	■	■
Microbat *	*Myotis lucifugus*	■	■	■	■	■	■		■	■	■
Minke whale *	*Balaenoptera acutorostrata*	■	■	■	■	■	■	■	■	■	■
Mouse	*Mus musculus*	■	■	■	■	■	■	■	■	■	■
Olive Baboon	*Papio anubis*	■	■	■	■	■	■	■	■	■	■
Opossum	*Monodelphis domestica*	■	■	■	■	■	■	■	■	■	■
Orangutan	*Pongo abelii*	■	■	■	■	■	■	■	■	■	■
Pig	*Sus scrofa*	■	■	■	■	■	■	■	■	■	■
Rabbit	*Oryctolagus cuniculus*		■	■	■	■	■	■	■	■	■
Rat	*Rattus norvegicus*	■	■	■	■	■	■	■	■	■	■
Rhinoceros	*Ceratotherium simum simum*	■	■	■	■	■	■	■	■	■	■
Sheep	*Ovis aries*	■		■	■	■	■	■	■	■	■
Shrew	*Sorex araneus*		■	■	■	■	■		■	■	■
Sperm whale *	*Physeter catodon*	■	■	■				■	■		
Squirrel	*Ictidomys tridecemlineatus*	■	■	■		■	■	■	■	■	
Walrus *	*Odobenus rosmarus*	■	■	■	■	■	■	■	■	■	■
Weddel seal *	*Leptonychotes weddellii*	■	■		■	■	■	■	■	■	■

AVES

Common Name	*Scientific Name*	*ATP2C2*	*CMIP*	*CNTNAP2*	*DCDC2*	*DYX1C1*	*FOXP2*	*KIAA0319*	*NFXL1*	*ROBO1*	*ROBO2*
Adelie Penguin	*Pygoscelis adeliae*	■	■	■	■	■	■	■	■	■	■
American Crow	*Corvus brachyrhynchos*	■	■	■	■	■	■	■	■	■	■
Anna's Hummingbird *	*Calypte anna*	■	■	■	■	■	■	■	■	■	■
Bald eagle	*Haliaeetus leucocephalus*	■	■	■	■	■	■	■	■	■	■
Budgerigar *	*Melopsittacus undulatus*	■	■	■	■	■	■	■	■	■	■
Chicken	*Gallus gallus*	■	■	■	■	■	■	■	■	■	■
Chimney swift	*Chaetura pelagica*	■	■		■	■	■	■	■	■	■
Collared flycatcher	*Ficedula albicollis*	■	■	■	■	■	■	■	■	■	■
Common cuckoo	*Cuculus canorus*	■	■	■	■	■	■	■	■	■	■
Common ostrich	*Struthio camelus*	■	■	■	■	■	■	■	■	■	■
Crested Ibis	*Nipponia nippon*	■	■	■	■	■	■	■	■	■	■
Downy Woodpecker	*Picoides pubescens*	■	■	■	■	■	■	■	■	■	■
Emperor penguin	*Aptenodytes forsteri*	■	■	■	■	■	■	■	■	■	■
Golden-collared Manakin	*Manacus vitellinus*	■	■	■	■	■	■	■	■	■	
Hoatzin	*Opisthocomus hoazin*	■	■	■	■	■	■	■	■	■	■
Kea (*)	*Nestor notabilis*	■	■		■	■		■	■		
Killdeer	*Charadrius vociferus*	■	■	■	■	■	■	■	■	■	■
Little Egret	*Egretta garzetta*	■	■	■	■	■	■	■	■	■	■
Mallard duck	*Anas platyrhynchos*	■	■	■	■	■	■	■	■		
Medium ground finch (*)	*Geospiza fortis*	■	■	■	■	■	■	■	■	■	■
Peregrine falcon	*Falco peregrinus*	■	■	■	■	■	■	■	■	■	■
Rock pigeon	*Columba livia*	■	■	■	■	■	■	■	■	■	■
Saker falcon	*Falco cherrug*	■	■	■	■	■	■	■	■	■	■
Tibetan ground-jay (*)	*Pseudopodoces humilis*	■	■	■	■	■	■	■	■	■	■
Turkey	*Meleagris gallopavo*	■	■	■	■	■	■	■	■	■	■
White troated sparrow*	*Zonotrichia albicollis*	■	■	■	■	■	■	■	■	■	
Zebra finch *	*Taeniopygia guttata*	■	■	■	■	■	■	■	■	■	■

Notes: Known * or suggested (*) "vocal learners" species. For each gene analyzed species are highlighted in cyan

Supplementary Table S2. Recombination breakpoints and accession numbers of the human and chicken sequences, used as reference for mammals and aves.

MAMMALS		
**Gene**	**Recombination Breakpoint**	**Gene ID (*Homo sapiens*)**
ATP2C2	0	NM_014861
CMIP<br>*Reg 1 (1-484aa)*<br>*Reg 2 (485-773aa)*	1	NM_198390
CNTNAP2<br>*Reg 1 (1-699aa)*<br>*Reg 2 (700-1331aa)*	1	NM_014141
DCDC2<br>*Reg 1 (1-268aa)*<br>*Reg 2 (269-476aa)*	1	NM_001195610
DYX1C1	0	NM_130810
FOXP2<br>*Reg 1 (1-152aa)*<br>*Reg 2 (153-194aa)*<br>*Reg 3 (195-715aa)*	2	NM_014491
KIAA0319	0	NM_001168374
NFXL1	0	NM_152995
ROBO1	0	NM_002941
ROBO2	0	NM_002942
**AVES**		
**Gene**	**Recombination Breakpoint**	**Gene ID (*Gallus gallus*)**
ATP2C2	0	XM_004944264
CMIP	0	XM_423491
CNTNAP2	0	XM_004935059
DCDC2<br>*Reg 1 (1-180aa)*<br>*Reg 2 (181-497aa)*	1	XM_003640752
DYX1C1	0	NM_001008674
FOXP2	0	JN677530
KIAA0319	0	ENSGALT00000020664
NFXL1	0	XM_003641238
ROBO1	0	XM_004938344
ROBO2	0	XM_416674

Supplementary Table S3. Likelihood ratio test (LRT) statistics for models of variable selective pressure among sites (codon frequency: F61).

Genes	Model[a]	− 2ΔlnL[b]	*p* Value (Bonferroni corrected)
**MAMMALS**			
ATP2C2	M8a vs M8	4.380	3.637×10^{-2}
	M7 vs M8	7.640	2.193×10^{-2}
CNTNAP2 (reg2)	M8a vs M8	19.365	1.080×10^{-5} (2.159×10^{-5})
	M7 vs M8	12.541	1.891×10^{-3} (3.782×10^{-3})
DYX1C1	M8a vs M8	7.880	4.997×10^{-3}
	M7 vs M8	14.770	6.203×10^{-4}
NFXL1	M8a vs M8	6.461	1.103×10^{-2}
	M7 vs M8	54.309	1.610×10^{-12}
ROBO2	M8a vs M8	4.587	3.222×10^{-2}
	M7 vs M8	24.946	3.828×10^{-6}
**AVES**			
**Genes**	**Model[a]**	**− 2ΔlnL[b]**	***p* Value (Bonferroni corrected)**
ATP2C2	M8a vs M8	9.628	1.916×10^{-3}
	M7 vs M8	12.572	1.862×10^{-3}
DCDC2 (reg2)	M8a vs M8	6.853	8.851×10^{-3} (1.770×10^{-2})
	M7 vs M8	13.882	9.672×10^{-4} (1.934×10^{-3})
FOXP2	M8a vs M8	14.740	6.299×10^{-4}
	M7 vs M8	12.074	5.113×10^{-4}
NFXL1	M8a vs M8	10.742	1.047×10^{-3}
	M7 vs M8	29.336	4.263×10^{-7}

Notes:
a: M7 is a null model that assumes that 0<dN/dS <1 is beta distributed among sites; M8 (positive selection model) is the same as M7 but also includes an extra category of sites with dN/dS >1. M8a is the same as M8, except that the 11th category cannot allow positive selection, but only neutral evolution.
b: 2ΔlnL is twice the difference of the natural logs of the maximum likelihood of the models being compared.

Supplementary Table S4. Positively selected sites in the human, chimpanzee and gorilla lineages.

Gene	Lineage	Codon	Derived AA	Pr[a]	Other methods[b]	SNPs
DCDC2						
	Gorilla	296	Thr	0,955		
		299	Ser	0,897	MEME	
DYX1C1						
	Gorilla	16	Val	0,880		
		174	Glu	0,974		
FOXP2						
	Human	303	Asn	0,965	MEME	
		325	Ser	0,969		
KIAA0319						
	Human	13	Arg	0,907		
		190	Val	0,851		
		306	Glu	0,970		
		327	Thr	0,971		
		364	Asn	0,945		
		765	Val	0,830		rs2817191
		865	Arg	0,861		
	Gorilla	739	Glu	0,781		
ROBO1						
	Chimpanzee	1134	Gln	0,811		
		1514	Val	0,813	MEME	

a. Posterior probability of γ≥ 1 as detected by gammaMap.
b Other methods that identified the same codon as positively selected

Supplementary Table S5. Candidate targets of Positive selection in human populations.

Gene	SNP ID	Genomic Position (GRCh37/hg19)	Derived Allele	DAF[a]			DIND rank (population)	F_{ST} rank (comparison)	DH Rank[b] (population)	Neanderthal Genotype	Denisovan Genotype	Notes[c]
				YRI	CEU	CHB						
CNTNAP2												
	rs802563	chr7:145955236	C	0.21	0.95	0.97	>0.99 (CEU)	>0.99 (CEU/YRI)	<0.01 (CEU)	G/G	G/G	modern-human-specific allele
	rs802566	chr7:145955882	C	0.03	0.95	0.97	>0.99 (CEU)	>0.99 (CEU/YRI)	<0.01 (CEU)	G/G	G/G	modern-human-specific allele
	rs1091835	chr7:145956972	G	0.03	0.95	0.97	>0.99 (CEU)	>0.99 (CEU/YRI)	<0.01 (CEU)	C/C	C/C	modern-human-specific allele
	rs2462818	chr7:145957082	G	0.17	0.95	0.97	>0.99 (CEU)	>0.99 (CEU/YRI)	<0.01 (CEU)	C/C	C/C	modern-human-specific allele
	rs813908	chr7:145957699	A	0.21	0.95	0.97	>0.99 (CEU)	>0.99 (CEU/YRI)	<0.01 (CEU)	T/T	T/T	modern-human-specific allele
	rs802567	chr7:145958559	T	0.15	0.96	0.98	>0.99 (CEU)	>0.99 (CEU/YRI)	<0.01 (CEU)	A/A	A/A	modern-human-specific allele
	rs802569	chr7:145960943	T	0.17	0.96	0.98	>0.99 (CEU)	>0.99 (CEU/YRI)	<0.01 (CEU)	C/C	C/C	modern-human-specific allele
	rs802571	chr7:145962185	A	0.17	0.96	0.98	>0.99 (CEU)	>0.99 (CEU/YRI)	N.S.	G/G	G/G	modern-human-specific allele
	rs802558	chr7:145967379	G	0.17	0.96	0.98	0.99 (CEU)	>0.99 (CEU/YRI)	N.S.	C/C	C/C	modern-human-specific allele
	rs10262823	chr7:145993677	A	0.14	0.95	0.98	>0.99 (CEU)	0.99 (CEU/YRI)	<0.01 (CEU)	G/G	G/G	modern-human-specific allele
	rs4615478	chr7:145998616	G	0.14	0.95	0.98	>0.99 (CEU)	0.99 (CEU/YRI)	<0.01 (CEU)	C/C	C/C	modern-human-specific allele
	rs10255169	chr7:146018519	G	0.05	0.94	0.96	>0.99 (CEU)<br>>0.99 (CHB)	>0.99 (CEU/YRI)<br>>0.99 (CHB/YRI)	<0.01 (CEU)<br><0.01 (CHB)	C/C	C/C	modern-human-specific allele
	rs7778737	chr7:146025508	G	0.24	0.98	0.96	0.99 (CHB)	0.99 (CHB/YRI)	N.S.	A/A	A/A	modern-human-specific allele
	rs7781275	chr7:146027319	A	0.09	0.95	0.96	0.99 (CEU)<br>0.99 (CHB)	>0.99 (CEU/YRI)<br>>0.99 (CHB/YRI)	N.S.	G/G	G/G	modern-human-specific allele
	rs7794540	chr7:146034095	C	0.08	0.94	0.96	0.99 (CEU)<br>0.99 (CHB)	>0.99 (CEU/YRI)<br>>0.99 (CHB/YRI)	<0.01 (CEU)<br><0.01 (CHB)	A/A	A/A	modern-human-specific allele
	rs7808617	chr7:146037098	G	0.38	0.97	0.97	>0.99 (CHB)	0.99 (CHB/YRI)	<0.01 (CHB)	G/G	G/G	
	rs7805444	chr7:146037517	A	0.22	0.92	0.97	0.99 (CHB)	0.99 (CHB/YRI)	<0.01 (CHB)	G/G	G/G	modern-human-specific allele
	rs17170043	chr7:146037998	T	0.22	0.92	0.97	0.99 (CHB)	0.99 (CHB/YRI)	<0.01 (CHB)	G/G	G/G	modern-human-specific allele
	rs17170044	chr7:146038198	T	0.22	0.92	0.97	0.99 (CHB)	0.99 (CHB/YRI)	<0.01 (CHB)	C/C	C/C	modern-human-specific

											allele
rs7791697	chr7:146038855	G	0.22	0.92	0.97	0.99 (CHB)	0.99 (CHB/YRI)	<0.01 (CHB)	T/T	T/T	modern-human-specific allele
rs10282451	chr7:146040701	G	0.22	0.92	0.97	0.99 (CHB)	0.99 (CHB/YRI)	<0.01 (CHB)	T/T	T/T	modern-human-specific allele
rs7807111	chr7:146041413	C	0.22	0.92	0.97	0.99 (CHB)	0.99 (CHB/YRI)	<0.01 (CHB)	G/G	G/G	modern-human-specific allele
rs344448	chr7:146402911	A	0.11	0.95	0.88	>0.99 (CEU)	>0.99 (CEU/YRI)	<0.01 (CEU)	C/C	C/C	modern-human-specific allele
rs28716585	chr7:146405742	T	0.11	0.95	0.88	>0.99 (CEU)	>0.99 (CEU/YRI)	<0.01 (CEU)	C/C	C/C	modern-human-specific allele
rs344478	chr7:146410467	A	0.01	0.95	0.88	0.99 (CEU)	>0.99 (CEU/YRI)	N.S.	C/C	C/C	modern-human-specific allele
rs344461	chr7:146422409	G	0	0.85	0.88	>0.99 (CEU)	>0.99 (CEU/YRI)	N.S.	A/A	A/A	modern-human-specific allele
rs189364	chr7:146423246	C	0.04	0.85	0.88	>0.99 (CEU)	>0.99 (CEU/YRI)	N.S.	G/G	G/G	modern-human-specific allele
rs2533090	chr7:146424393	A	0.04	0.85	0.88	>0.99 (CEU)	>0.99 (CEU/YRI)	N.S.	T/T	T/T	modern-human-specific allele
rs10239811	chr7:146501345	T	0.16	0.99	0.98	>0.99 (CEU)	>0.99 (CEU/YRI)	<0.01 (CEU)	C/C	C/C	modern-human-specific allele
rs10229442	chr7:146503039	C	0.16	0.99	0.98	>0.99 (CEU)	>0.99 (CEU/YRI)	<0.01 (CEU)	T/T	T/T	modern-human-specific allele
rs7776949	chr7:146505140	T	0.2	0.99	0.98	>0.99 (CEU)	>0.99 (CEU/YRI)	<0.01 (CEU)	G/G	G/G	modern-human-specific allele
rs58702770	chr7:146507701	A	0.16	0.99	0.98	>0.99 (CEU)	>0.99 (CEU/YRI)	N.S.	G/G	G/G	modern-human-specific allele
rs61197728	chr7:146512417	A	0.2	0.99	0.98	>0.99 (CEU)	>0.99 (CEU/YRI)	<0.01 (CEU)	G/G	G/G	modern-human-specific allele
rs6962773	chr7:146514125	T	0.16	0.99	0.98	>0.99 (CEU)	>0.99 (CEU/YRI)	<0.01 (CEU)	A/A	A/A	modern-human-specific allele
rs7805995	chr7:146516465	A	0.2	0.99	0.98	>0.99 (CEU)	>0.99 (CEU/YRI)	<0.01 (CEU)	G/G	G/G	modern-human-specific allele
rs7806453	chr7:146516748	A	0.2	0.99	0.98	>0.99 (CEU)	>0.99 (CEU/YRI)	<0.01 (CEU)	C/C	C/C	modern-human-specific allele
rs11982965	chr7:146521688	A	0.24	0.99	0.98	>0.99 (CEU)	>0.99 (CEU/YRI)	N.S.	G/G	G/G	modern-human-specific allele
rs73459353	chr7:146530086	T	0.16	0.99	0.98	>0.99 (CEU)	>0.99 (CEU/YRI)	<0.01 (CEU)	C/C	C/C	modern-human-specific allele
rs60667967	chr7:146532320	G	0.28	0.99	0.98	>0.99 (CEU)	>0.99 (CEU/YRI)	<0.01 (CEU)	T/T	T/T	modern-human-specific allele
rs10268245	chr7:146533525	C	0.19	0.99	0.98	>0.99 (CEU)	>0.99 (CEU/YRI)	<0.01 (CEU)	T/T	T/T	modern-human-specific allele
rs9691764	chr7:146534157	A	0.16	0.99	0.98	>0.99 (CEU)	>0.99 (CEU/YRI)	<0.01 (CEU)	C/C	C/C	modern-human-specific allele

rs35118410	chr7:147070723	C	0	0.27	0.01	0.99 (CEU)	>0.99 (CEU/YRI)<br>0.99 (CEU/CHB)	N.S.	G/G	G/G	modern-human-specific allele
rs17498276	chr7:147073510	C	0	0.27	0.01	0.99 (CEU)	>0.99 (CEU/YRI)<br>0.99 (CEU/CHB)	N.S.	T/T	T/T	modern-human-specific allele
rs1528520	chr7:147182348	G	0	0.28	0.01	>0.99 (CEU)	>0.99 (CEU/YRI)<br>>0.99 (CEU/CHB)	N.S.	A/A	A/A	modern-human-specific allele
rs17224658	chr7:147193582	T	0	0.28	0.01	>0.99 (CEU)	>0.99 (CEU/YRI)<br>>0.99 (CEU/CHB)	N.S.	C/C	C/C	modern-human-specific allele
rs12703922	chr7:147194326	A	0	0.28	0.01	>0.99 (CEU)	>0.99 (CEU/YRI)<br>>0.99 (CEU/CHB)	N.S.	G/G	G/A	
rs12703923	chr7:147194459	C	0	0.28	0.01	>0.99 (CEU)	>0.99 (CEU/YRI)<br>>0.99 (CEU/CHB)	N.S.	T/T	T/T	modern-human-specific allele
rs112441834	chr7:147364931	T	0.89	0.93	1	>0.99 (YRI)	>0.99 (CHB/YRI)	<0.01 (YRI)	C/C	C/C	modern-human-specific allele
rs7786677	chr7:147364957	G	0.89	0.93	1	>0.99 (YRI)	>0.99 (CHB/YRI)	<0.01 (YRI)	A/A	A/A	modern-human-specific allele
rs75743142	chr7:147365348	G	0.89	0.93	1	>0.99 (YRI)	>0.99 (CHB/YRI)	<0.01 (YRI)	A/A	A/A	modern-human-specific allele*
rs80148568	chr7:147365554	A	0.92	0.93	1	>0.99 (YRI)	>0.99 (CHB/YRI)	<0.01 (YRI)	G/G	G/G	modern-human-specific allele*
rs851727	chr7:147366953	T	0.61	0.91	1	>0.99 (CEU)	>0.99 (CEU/CHB)	<0.01 (CEU)	C/C	C/C	modern-human-specific allele
rs74806085	chr7:147367929	C	0.92	0.93	1	>0.99 (YRI)<br>0.99 (CEU)	>0.99 (CHB/YRI)<br>>0.99 (CEU/CHB)	<0.01 (YRI)<br><0.01 (CEU)	A/A	C/A	
rs76297534	chr7:147367933	G	0.92	0.93	1	>0.99 (YRI)<br>0.99 (CEU)	>0.99 (CHB/YRI)<br>>0.99 (CEU/CHB)	<0.01 (YRI)<br><0.01 (CEU)	T/T	G/T	*
rs78759472	chr7:147426830	C	0.31	0	0	0.99 (YRI)	>0.99 (CEU/YRI)<br>>0.99 (CHB/YRI)	N.S.	T/T	T/T	modern-human-specific allele
rs57678081	chr7:147427187	A	0.31	0	0	0.99 (YRI)	>0.99 (CEU/YRI)<br>>0.99 (CHB/YRI)	N.S.	T/T	T/T	modern-human-specific allele
rs7457355	chr7:147432208	T	0.29	0	0	>0.99 (YRI)	>0.99 (CEU/YRI)<br>>0.99 (CHB/YRI)	N.S.	G/G	-	modern-human-specific allele
rs2177722	chr7:147438725	A	0.31	0	0	0.99 (YRI)	>0.99 (CEU/YRI)<br>>0.99 (CHB/YRI)	N.S.	T/T	T/T	modern-human-specific allele
rs6954961	chr7:147442296	T	0.32	0	0	>0.99 (YRI)	>0.99 (CEU/YRI)<br>>0.99 (CHB/YRI)	N.S.	C/C	C/C	modern-human-specific allele
rs61089905	chr7:147443619	A	0.32	0	0	0.99 (YRI)	>0.99 (CEU/YRI)<br>>0.99 (CHB/YRI)	N.S.	C/C	C/C	modern-human-specific allele
rs17170666	chr7:147444440	G	0.32	0	0	0.99 (YRI)	>0.99 (CEU/YRI)<br>>0.99 (CHB/YRI)	N.S.	A/A	A/A	modern-human-specific allele
rs7799600	chr7:147445082	C	0.51	1	0.94	>0.99 (CHB)	>0.99 (CEU/CHB)	<0.01 (CHB)	T/T	T/T	modern-human-specific

												allele
	rs10952709	chr7:147449538	G	0.55	1	0.94	>0.99 (CHB)	>0.99 (CEU/CHB)	<0.01 (CHB)	A/A	A/A	modern-human-specific allele
	rs12155395	chr7:147449615	C	0.51	1	0.94	0.99 (CHB)	>0.99 (CEU/CHB)	<0.01 (CHB)	G/G	G/G	modern-human-specific allele
	rs144946067	chr7:147466612	A	0.98	1	0.97	>0.99 (YRI)	>0.99 (CEU/YRI)	<0.01 (YRI)	G/G	G/G	modern-human-specific allele*
	rs7457261	chr7:147466675	C	0.98	1	0.97	>0.99 (YRI)	>0.99 (CEU/YRI)	<0.01 (YRI)	G/G	G/G	modern-human-specific allele*
	rs12538275	chr7:147477023	G	0.98	1	0.97	>0.99 (YRI)	>0.99 (CEU/YRI)	<0.01 (YRI)	T/T	G/G	
FOXP2												
	rs73436138	chr7:114129502	C	0.89	1	1	0.99 (YRI)	>0.99 (CEU/YRI) >0.99 (CHB/YRI)	N.S.	T/T	T/T	modern-human-specific allele*
	rs113503202	chr7:114152973	C	0.88	1	1	>0.99 (YRI)	>0.99 (CEU/YRI) >0.99 (CHB/YRI)	N.S.	T/T	T/T	modern-human-specific allele
	rs977429	chr7:114153102	T	0.88	1	1	>0.99 (YRI)	>0.99 (CEU/YRI) >0.99 (CHB/YRI)	N.S.	C/C	C/C	modern-human-specific allele*
	rs111591821	chr7:114156793	A	0.88	1	1	>0.99 (YRI)	>0.99 (CEU/YRI) >0.99 (CHB/YRI)	N.S.	C/C	C/C	modern-human-specific allele
	rs73429321	chr7:114157605	G	0.88	1	1	>0.99 (YRI)	>0.99 (CEU/YRI) >0.99 (CHB/YRI)	N.S.	C/C	C/C	modern-human-specific allele*
	rs17137077	chr7:114158476	T	0.88	0.99	1	>0.99 (YRI)	>0.99 (CHB/YRI)	N.S.	A/A	A/A	modern-human-specific allele*
ROBO1												
	rs4680920	chr3:79066834	G	0.91	1	1	>0.99 (YRI)	>0.99 (CEU/YRI) >0.99 (CHB/YRI)	N.S.	T/T	T/T	modern-human-specific allele*
	rs6548608	chr3:79073792	G	0.92	1	1	>0.99 (YRI)	>0.99 (CEU/YRI) >0.99 (CHB/YRI)	<0.01 (YRI)	A/A	A/A	modern-human-specific allele*
	rs1374882	chr3:79075348	G	0.9	1	1	>0.99 (YRI)	>0.99 (CEU/YRI) >0.99 (CHB/YRI)	<0.01 (YRI)	A/A	A/A	modern-human-specific allele
	rs1349022	chr3:79075910	C	0.92	1	1	>0.99 (YRI)	>0.99 (CEU/YRI) >0.99 (CHB/YRI)	<0.01 (YRI)	G/G	G/G	modern-human-specific allele*
	rs6795076	chr3:79089397	G	0.9	0.99	1	>0.99 (YRI)	>0.99 (CHB/YRI)	N.S.	A/A	A/A	modern-human-specific allele*
	rs7629953	chr3:79093338	T	0.88	0.99	1	>0.99 (YRI)	>0.99 (CHB/YRI)	<0.01 (YRI)	G/G	G/G	modern-human-specific allele*
	rs7639472	chr3:79214616	A	0.68	0.94	1	0.99 (CEU)	>0.99 (CEU/CHB)	<0.01 (CEU)	C/C	C/C	modern-human-specific allele
	rs76930654	chr3:79219102	G	0.68	0.94	1	>0.99 (CEU)	>0.99 (CEU/CHB)	<0.01 (CEU)	A/A	A/A	modern-human-specific allele
	rs74494901	chr3:79224596	C	0.68	0.94	1	>0.99 (CEU)	>0.99 (CEU/CHB)	<0.01 (CEU)	T/T	T/T	modern-human-specific allele

rs79695773	chr3:79228025	T	0.64	0.94	1	>0.99 (CEU)	>0.99 (CEU/CHB)	<0.01 (CEU)	C/C	C/C	modern-human-specific allele
rs77283623	chr3:79229019	C	0.68	0.94	1	>0.99 (CEU)	>0.99 (CEU/CHB)	<0.01 (CEU)	T/T	T/T	modern-human-specific allele
rs4405871	chr3:79229918	T	0.78	0.94	1	>0.99 (CEU)	>0.99 (CEU/CHB)	<0.01 (CEU)	C/C	C/C	modern-human-specific allele*
rs77658577	chr3:79230203	T	0.64	0.94	1	>0.99 (CEU)	>0.99 (CEU/CHB)	<0.01 (CEU)	A/A	A/A	modern-human-specific allele
rs76692280	chr3:79235072	A	0.55	0.94	1	0.99 (CEU)	>0.99 (CEU/CHB)	<0.01 (CEU)	C/C	C/C	modern-human-specific allele
rs716681	chr3:79236284	C	0.55	0.94	1	0.99 (CEU)	>0.99 (CEU/CHB)	<0.01 (CEU)	T/T	T/T	modern-human-specific allele
rs1563382	chr3:79236890	A	0.07	0.93	0.99	0.99 (CEU)	>0.99 (CEU/YRI)	<0.01 (CEU)	G/G	G/G	modern-human-specific allele
rs76223368	chr3:79236962	G	0.68	0.94	1	0.99 (CEU)	>0.99 (CEU/CHB)	<0.01 (CEU)	A/A	A/A	modern-human-specific allele
rs78729468	chr3:79237054	C	0.68	0.94	1	0.99 (CEU)	>0.99 (CEU/CHB)	<0.01 (CEU)	T/T	T/T	modern-human-specific allele
rs10511123	chr3:79240461	A	0.64	0.94	1	0.99 (CEU)	>0.99 (CEU/CHB)	<0.01 (CEU)	T/T	T/T	modern-human-specific allele
rs11920038	chr3:79241779	G	0.64	0.94	1	0.99 (CEU)	>0.99 (CEU/CHB)	<0.01 (CEU)	C/C	C/C	modern-human-specific allele
rs77770813	chr3:79248466	C	0.64	0.94	1	>0.99 (CEU)	>0.99 (CEU/CHB)	<0.01 (CEU)	A/A	A/A	modern-human-specific allele
rs11920736	chr3:79513399	A	0.81	0.99	0.99	0.99 (YRI)	>0.99 (CHB/YRI)	<0.01 (YRI)	A/A	G/G	*
rs78123674	chr3:79513699	G	0.81	0.99	0.99	0.99 (YRI)	>0.99 (CHB/YRI)	<0.01 (YRI)	G/G	A/A	*
rs76483894	chr3:79514084	A	0.81	0.99	0.99	0.99 (YRI)	>0.99 (CHB/YRI)	<0.01 (YRI)	A/A	G/G	*
rs78726247	chr3:79514998	G	0.81	0.99	0.99	0.99 (YRI)	>0.99 (CHB/YRI)	<0.01 (YRI)	G/G	A/A	*
ROBO2											
rs62251837	chr3:77257832	T	0.96	0.95	1	0.99 (YRI)	>0.99 (CHB/YRI)	<0.01 (YRI)	C/C	T/C	*
rs62251858	chr3:77267679	C	0.97	0.95	1	0.99 (YRI)	>0.99 (CHB/YRI)	<0.01 (YRI)	C/C	T/C	
rs60679691	chr3:77268414	A	0.97	0.95	1	0.99 (YRI)	>0.99 (CHB/YRI)	N.S.	A/A	A/A	
rs62253267	chr3:77269336	A	0.97	0.95	1	>0.99 (YRI)	>0.99 (CHB/YRI)	N.S.	T/T	T/T	modern-human-specific allele
rs62253278	chr3:77277795	C	0.96	0.95	1	0.99 (YRI)	>0.99 (CHB/YRI)	N.S.	G/G	C/G	
rs62253280	chr3:77278158	C	0.96	0.95	1	0.99 (YRI)	>0.99 (CHB/YRI)	N.S.	A/A	C/A	*

[a]Derived Allele Frequency

[b]Rank percentile relative to the genomic window(s) in which the SNP falls (see text for details); N.S.: Not Significant

[c] Modern-human-specific allele indicates a SNP where the Denisovan and the Altai Neanderthal are homozygous for the ancestral allele regardless of its DAF in the human populations, whereas the * indicates a SNP defined as modern-human-specific site as defined by Prufer, *et al.* (2014). The complete genome sequence of a Neanderthal from the Altai Mountains. *Nature* 505, 43-49.
